# Positively selected amino acid replacements within the RuBisCO enzyme of oak trees are associated with ecological adaptations

**DOI:** 10.1371/journal.pone.0183970

**Published:** 2017-08-31

**Authors:** Carmen Hermida-Carrera, Mario A. Fares, Ángel Fernández, Eustaquio Gil-Pelegrín, Maxim V. Kapralov, Arnau Mir, Arántzazu Molins, José Javier Peguero-Pina, Jairo Rocha, Domingo Sancho-Knapik, Jeroni Galmés

**Affiliations:** 1 Research Group on Plant Biology under Mediterranean Conditions, Universitat de les Illes Balears-INAGEA, Palma, Balearic Islands, Spain; 2 Integrative and Systems Biology Group, Department of Abiotic Stress, Instituto de Biología Molecular y Celular de Plantas (CSIC–UPV), Valencia, Spain; 3 Department of Genetics, University of Dublin, Trinity College Dublin, Dublin 2, Ireland; 4 Unidad de Recursos Forestales, C.I.T.A. de Aragón, Zaragoza, Spain; 5 School of Natural and Environmental Sciences, Newcastle University, Newcastle-Upon-Tyne, United Kingdom; 6 Computational Biology and Bioinformatics Research Group, Department of Mathematics and Computer Science, Universitat de les Illes Balears, Palma, Balearic Islands, Spain; National Cheng Kung University, TAIWAN

## Abstract

Phylogenetic analysis by maximum likelihood (PAML) has become the standard approach to study positive selection at the molecular level, but other methods may provide complementary ways to identify amino acid replacements associated with particular conditions. Here, we compare results of the decision tree (DT) model method with ones of PAML using the key photosynthetic enzyme RuBisCO as a model system to study molecular adaptation to particular ecological conditions in oaks (*Quercus*). We sequenced the chloroplast *rbcL* gene encoding RuBisCO large subunit in 158 *Quercus* species, covering about a third of the global genus diversity. It has been hypothesized that RuBisCO has evolved differentially depending on the environmental conditions and leaf traits governing internal gas diffusion patterns. Here, we show, using PAML, that amino acid replacements at the residue positions 95, 145, 251, 262 and 328 of the RuBisCO large subunit have been the subject of positive selection along particular *Quercus* lineages associated with the leaf traits and climate characteristics. In parallel, the DT model identified amino acid replacements at sites 95, 219, 262 and 328 being associated with the leaf traits and climate characteristics, exhibiting partial overlap with the results obtained using PAML.

## Introduction

RuBisCO is one of the best-studied enzymes and is often used as a model protein in evolutionary studies. During photosynthesis, RuBisCO binds CO_2_ to the Calvin cycle intermediate ribulose-1,5-bisphosphate (RuBP), thereby acting as the essential entry point for carbon into the biosphere. Due to its imperfect ability to distinguish between CO_2_ and O_2_, RuBisCO also catalyzes the oxygenation of RuBP, giving rise to the energy-dissipating process of photorespiration. Compared to other catalysts, RuBisCO is a sluggish enzyme, with a catalytic turnover rate (*k*_cat_^c^) of about 3 s^−1^ in terrestrial plants [1]. Alongside these catalytic imperfections and its large molecular weight, RuBisCO also represents a significant nitrogen investment, typically accounting for 25–30% of the leaf total nitrogen in C_3_ plants [2].

The photosynthetic process adapts to abiotic stress, such as high temperature or water deficit [3, 4, 5], by optimizing leaf conductance (stomatal and mesophyll) governing CO_2_ diffusion [6] and by adjustments in the activity and concentration of RuBisCO and other rate limiting enzymes [7, 8, 5]. Temperature and CO_2_ concentration at RuBisCO active sites are the main driving forces of RuBisCO evolution and adaptation [9, 10, 11, 12, 13, 14, 15]. Computational analysis of carbon uptake at the leaf [16] and canopy level [17] also suggests that optimization of RuBisCO kinetics in modern C_3_ plants depends on the temperature regime and CO_2_ concentration. Therefore, plants from dry environments and plants with high leaf mass per area have the lowest CO_2_ diffusion, and tend to have higher RuBisCO affinity for CO_2_ [12, 18]. By contrast, plants possessing the C_4_ carbon concentration mechanism have faster, but less CO_2_ specific RuBisCO [19, 20, 21, 22, 23, 24]. High temperatures decrease the ratio of CO_2_/O_2_ dissolved in the leaf liquid media, and directly decreases the affinity of RuBisCO for CO_2_ [25]. Accordingly, adaptation to higher temperatures can be achieved by a greater specificity of RuBisCO for CO_2_ (*S*_c/o_), thereby reducing the loss of carbon due to photorespiration. Selection pressure on RuBisCO with increased *S*_c/o_ in hot environments has been demonstrated in some thermophilic red algae [26] and in terrestrial plants [12]. Because of the trade-off between RuBisCO affinity for CO_2_ and maximum carboxylase activity (*k*_cat_^c^), the selection for increased affinity for CO_2_ would inevitably take place at the expense of decreased *k*_cat_^c^ [13, 14]. Such fine-tuning of RuBisCO kinetic traits is attributed to environmentally driven changes at the molecular level, most likely amino acid replacements within the catalytic large subunit.

In higher plants and green algae, the structure of RuBisCO consists of eight chloroplast-encoded large (L, 50–55 kDa) and eight nucleus-encoded small (S, 12–18 kDa) subunits assembled into a hexadecamer [27]. Large subunits possess the active site and therefore primarily determine RuBisCO kinetic traits [28], although recent studies demonstrate that S-subunits can also influence catalysis [29, 30, 31]. Directed mutagenesis and a variety of recombinant RuBisCOs from plastome-transformed plants allowed identifying molecular changes in L-subunit that translate into changes in RuBisCO catalysis, as well as determining how they affected photosynthesis and plant growth [32, 33, 34, 35, 36, 37]. Recent studies have demonstrated the relationship between amino acid polymorphism in the L-subunit of RuBisCO and catalytic efficiency in natural vegetation and crops, by comparing both distant phylogenetic lineages [18, 38, 39] and closely related species [15, 40] of land plants.

Studies comparing the rates of non-synonymous and synonymous substitutions along phylogenies have demonstrated that positive Darwinian selection is acting on RuBisCO within most lineages of plants, but is restricted to a relatively small number of residues [41, 42, 43, 44, 45, 46, 47, 48, 49]. Results derived from analyses of RuBisCO molecular adaptation complement trends in RuBisCO kinetics and confirm the predominant role of some environmental and physiological factors driving RuBisCO evolution. For example, signatures of positive selection are associated with changes in intracellular concentrations of CO_2_ driven by carbon-concentrating mechanisms, both in algae and terrestrial C_4_ plants [43, 48, 49, 50].

Mapping positively selected residues within the protein structure helps to locate catalytically important regions of RuBisCO, and suggests candidate amino acid replacements which could be implemented to optimize RuBisCO performance in crops [42, 48, 49]. However, the effect of an amino acid replacement on protein properties could vary in the presence of other mutations, either individually or together, because of the molecular sign epistasis among mutations [51]. These epistatic interactions impose strong selective constraints on amino acid replacements and also may explain the failure of most attempts to improve RuBisCO catalysis by single point mutations [34, 52]. In agreement with this prediction, positive selection analysis must also account for co-adaptive amino acid replacements through the identification of coevolutionary signatures to find how key residue changes affect RuBisCO structure and function. Coevolutionary studies have been applied to various proteins [53, 54, 55], but only recently to RuBisCO [47, 56]. It has been shown that coevolution of residues is common in RuBisCO of land plants and there is an overlap between coevolving and positively selected residues [56].

Evolutionary analyses are needed to identify adaptive changes in the Rubisco sequence, but the drivers of such evolution must also be investigated. In this paper, we used a predictive model called decision tree (DT), which is able to statistically associate a combination of environmental variables to variation in the amino acid residues. A DT can be used for both classification (classification tree) and regression (regression tree) tasks. We used this model for classification tasks, which are frequently employed in applied fields such as engineering and medicine [57, 58, 59, 60, 61]. A DT implicitly performs feature selection and requires relatively little effort for data preparation. The analysis is straightforward, the results shown graphically and they can be easily interpreted.

The objective of the study was to investigate molecular adaptation of *Quercus* RuBisCO to particular ecological conditions and to test if leaf morphological traits are associated with adaptive amino acid substitutions. To achieve this, we compared two different methodologies: the DT model and phylogenetic analysis by maximum likelihood. We selected oak (*Quercus*) species as a model group for this study because this genus contains a large number of species (*ca*. 500) inhabiting a wide range of environments. Both evergreen and deciduous oak species have contrasting leaf morphology [62], and therefore variable diffusive limitations to CO_2_ transfer from the atmosphere to the site of carboxylation [63]. Finally, oaks are often an ecosystem-defining species in most broad-leaved forests worldwide making them an ecologically important group.

## Materials and methods

### Taxon selection and sampling

A total of 174 species in Fagales were selected for the study (S1 Table). These species belong to the Fagaceae (*n* = 170) and Nothofagaceae (*n* = 4). Within Fagaceae, the majority of the species belong to *Quercus* (*n* = 158; ca. 30% of the total number of *Quercus* species).

Each species was classified according to its geographic distribution, prevalent climate and leaf habit (S1 Table). The geographic distribution area of each species was assigned according to Govaerts et al. (1998) [64] and information found in publicly available databases [65, 66, 67, 68]. The prevalent climate was obtained by overlapping the species geographical distribution in our study and the Köppen-Geiger world map of climate classification [69]. To simplify the analysis, fifteen Köppen-Geiger climate types were grouped into six: 1) tropical (including climates Af, Am and Aw according to Köppen-Geiger classification); 2) arid steppe (Bsh and Bsk); 3) temperate with dry winter and hot or warm summer (Cwa and Cwb); 4) temperate with dry summer and hot or warm summer (Csa and Csb); 5) temperate or cold without dry season and hot or warm summer (Cfa, Cfb, Dfa and Dfb) and 6) cold with dry summer and hot or warm summer (Dsa, andDsb) (S2 Table). Regarding the leaf habit, species were classified as evergreens (those species retaining their leaves during the whole year), deciduous (when losing all leaves during the unfavourable season) and semi-evergreen (those species that lose some leaves during the unfavourable season, depending on its length and severity).

Leaves from all species were sampled from living collections of Jardín Botánico de Iturrarán (Parque Natural de Pagoeta, Aya, Guipúzcoa, Spain), with the exceptions of *Q*. *palmeri*, *Q*. *baloot* and *Q*. *vaccinifolia*, which were collected from The Cheviton Barton collection (Bevon, UK). For each species, leaf density was calculated from leaf thickness and leaf mass area (LMA) measurements performed on fully expanded leaves that developed in the external part of the tree canopy (i.e., exposed to full solar irradiation). The leaf thickness of each species was measured on two discs (disc area = 0.33 cm^2^) per leaf from five fully hydrated leaves, collected from three to five different individuals. The leaf thickness was measured using a digital contact sensor GTH10L coupled to an amplifier GT-75AP (GT Series, Keyence Corporation, Japan) [70]. Afterwards, LMA of each disc was calculated as the ratio between the dry weight and the area. The dry weight was obtained after drying the leaf discs in a ventilated oven at 60°C until constant weight (typically after 2 days).

### DNA sequencing

Total genomic DNA was extracted from leaf material using the DNeasy Plant Mini Kit (Qiagen Ltd., Crawley, UK) according to the manufacturer’s protocol.

We sequenced chloroplast genes *rbcL* and *matK* [71, 72]. To obtain the full *rbcL* sequence (1428 nucleotides), the gene was amplified using primers esp2F (5´-AATTCATGAGTTGTAGGGAGGGACTT-3´) and 1494R (5´-GATTGGGCCGAGTTTAATTTAC-3´). The *matK* gene was amplified in 43 species using the primer X390_F (5´- CGATCTATTCATTCAATATTTC-3´) and Xmatk9_R (5´-CAATCATTCGTGATTGGCCAG -3´). For 42 species, we obtained the nuclear microsatellite loci (SSRs) from [73] (QmC00716, QmC01095, QmC01990, QmC02241) and from [74] (ssrQpZAG15, ssrQpZAG46, ssrQpZAG110, ssrQrZAG-7, ssrQrZAG-20).

All PCR reactions were performed using the BioMix Red reagent mix (Bioline Ltd., London, UK). The PCR program for the amplification of the *rbcL* comprised an initial denaturation at 95°C, 2 min, and 36 cycles of 93°C for 30 s, 53°C for 30 s and 72°C for 3.5 min, and a final extension at 72°C for 30 min. The PCR program for the amplification of the *matK* gene comprised an initial denaturation at 95°C for 2 min, followed by 35 cycles of 30 s at 94°C (denaturing), 45 s at the annealing temperature of 56°C, 2 min at 72°C (extension), and a final extension phase of 7 min at 72°C. The microsatellites were amplified using the following PCR conditions: 95°C for 2 min, and 35 cycles of 95°C for 30 s, 50°C for 30 s and 72°C for 2 min, and a final extension at 72°C for 5 min. The *rbcL* and *matK* PCR products were separated on 2% agarose gels buffered with 1X TAE and purified using Roche High Pure PCR Product Purification Kit (Roche Diagnostics Corporation P.O., Indiana, USA). Chloroplast gene sequencing was performed using an ABI 3130 Genetic analyzer with the ABI BigDyeTM Terminator Cycle Sequencing Ready Reaction Kit (Applied Biosystems, Foster City, California, USA). For microsatellites, we used the ABI 3130 XL Genetic analyzer and fragment analysis was performed using the GeneMapper software v4.1 (Applied Biosystems). The DNA sequences from the chloroplast markers were aligned using Clustal X [75] and manually adjusted with Bioedit v.7.2.5 [76]. All variable sites were checked against the original sequence chromatograms, and doubtful regions were sequenced again. All newly generated sequences were submitted to the GenBank (S3 Table).

### Phylogenetic analyses

**Fig 1:**
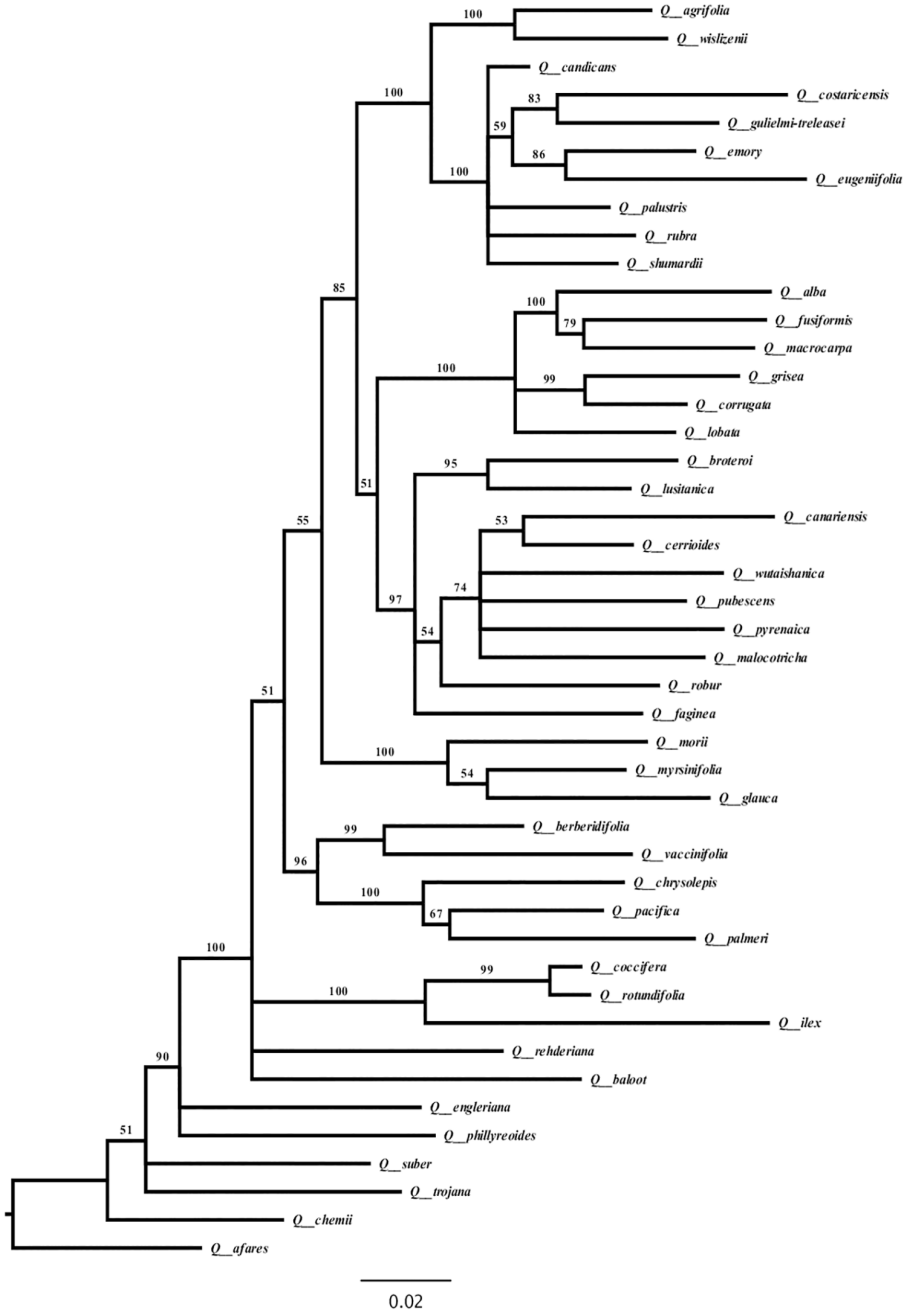
*Quercus* large dataset Bayesian phylogram based on 158 *rbcL* sequences. Numbers above branches correspond to Bayesian posterior probabilities. The figure was edited using FigTree Version 1.4.0 [77].

**Fig 2:**
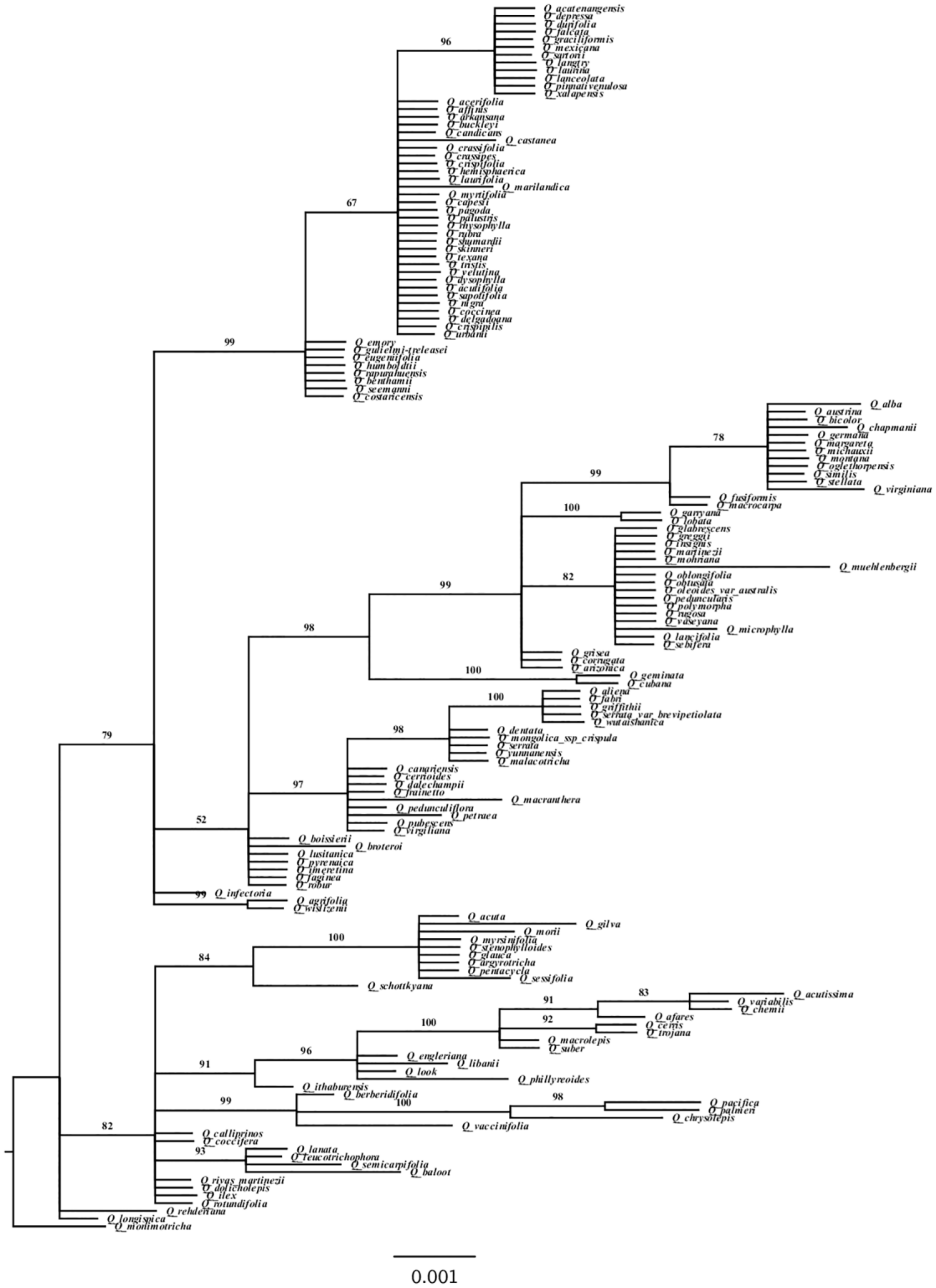
*Quercus* small dataset Bayesian phylogeny based on 45 sequences of *rbcL*, 43 *matK* and 42 microsatellites. Numbers above branches correspond to Bayesian posterior probabilities. The figure was edited using FigTree Version 1.4.0 [77].

**Fig 3:**
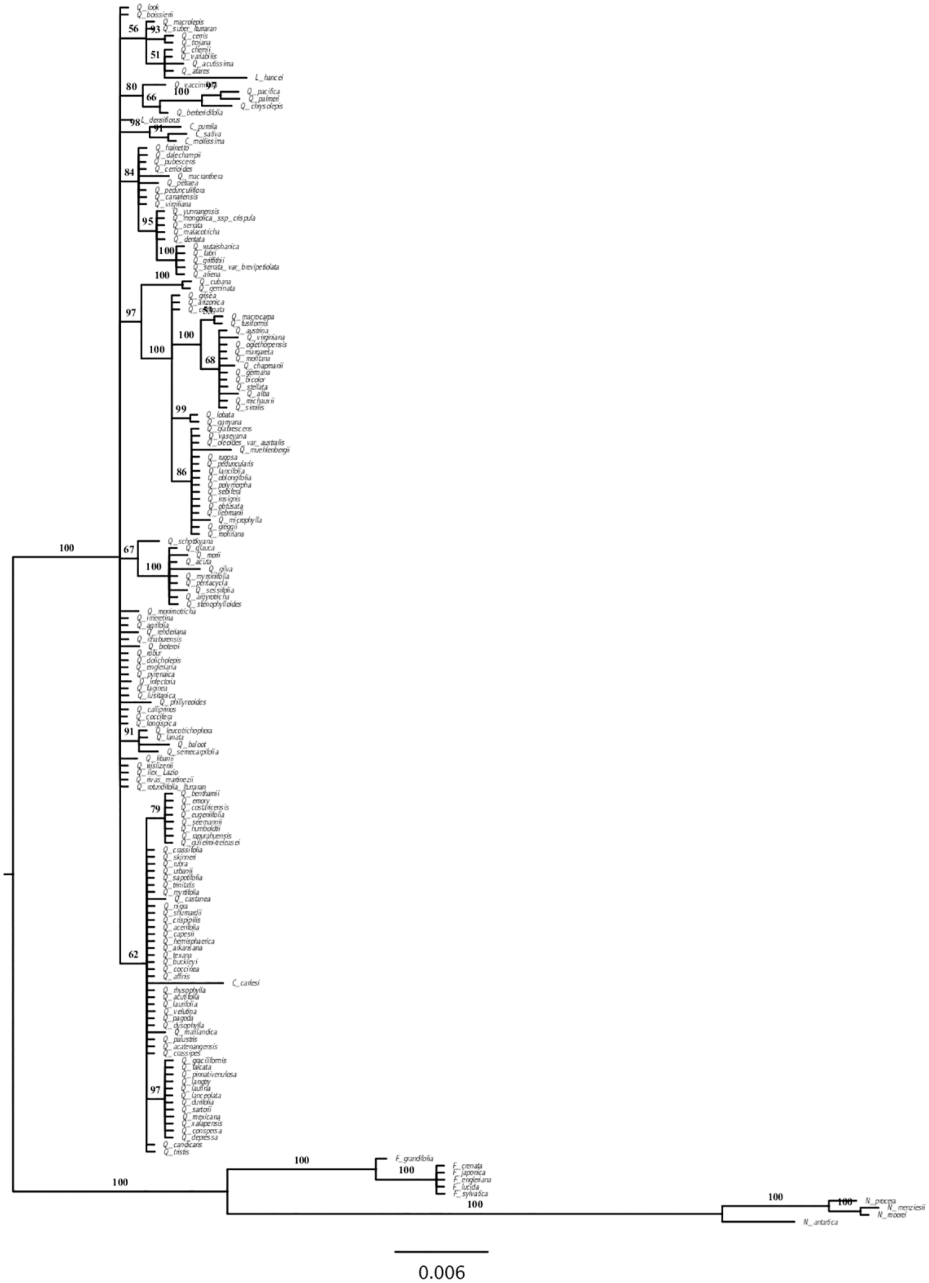
Fagales Bayesian phylogram based on 174 *rbcL* sequences. Numbers above branches correspond to Bayesian posterior probabilities. The figure was edited using FigTree Version 1.4.0 [77].

We inferred the phylogenetic relationships from the nucleotide data using Bayesian inference (BI). We constructed a phylogeny using *rbcL* sequences from the 158 *Quercus* species (denoted *Quercus* large dataset) (Fig 1). The tree topology was not fully resolved for this group when using only one gene. Because we require a robust phylogeny to detect adaptive evolution by maximum likelihood, we chose a subset of species to construct a multilocus tree with better-resolved topology. The tree was constructed with a concatenated alignment of 45 *rbcL*, 43 *matK* and 42 SSRs for *Quercus* species (denoted *Quercus* small dataset) (Fig 2). Tree topologies using *rbcL* were congruent with those based on the use of multiple genes, with both leading to similar lists of amino acid sites detected to have evolved under positive selection. Finally, we constructed the phylogeny for all 174 species containing the Fagaceae and Nothofagaceae species (denoted Fagales henceforward) (Fig 3).

Nucleotide sequences were translated into amino acid sequences with MEGA 5 software [78] and aligned online using MAFFT [79]. The optimal DNA substitution model was determined by Modeltest v.3.7 package [80, 81] by comparing available models using Bayesian information criterion (BIC). BI was performed in MrBayes version 3.2 [82] allowing different models for each region (*rbcL*, *matK* and SSRs). Markov Chain Monte Carlo (MCMC) used two independent runs of 1 × 10^6^ generations. Trees for *Quercus* small and Fagales datasets were sampled every 300 generations. For the *Quercus* large dataset the MCMC used independent runs of 5 × 10^6^ generations and trees were sampled every 100 generations. The first 25% of the runs were discarded as burn-in. The trees sampled before reaching a stable posterior probability (PP) were excluded from the consensus. A majority rule consensus of the remaining trees from the two runs was edited in FigTree v 1.4.0 [77] and used as BI tree.

### Maximum likelihood tests for positive selection

Six different codon based models were applied using the codeml program of the PAML package version 4.7 to test for the presence of positive selection [83]. These models were compared for goodness-of-fit to the data and phylogenies using the Likelhood Ratio Test (LRT) and the best model was used to estimate the nonsynonymous-to-synonymous rates ratio (ω = *d*_N_/*d*_S_). This ratio represents the selective pressures acting on the protein-coding gene with values of *ω* = 1, *ω* < 1, and positive *ω* > 1, being indicative of neutral evolution, purifying selection and positive selection, respectively. BI trees were used as the reference topologies for the PAML analyses (Figs 1–3).

Site models allow the *ω* ratio to vary among codons in the protein [84, 85]. Model M1a assumes the same selection pressures on all branches of the phylogenetic tree. In this model, codons can either evolve neutrally or under purifying selection, and thus the estimated values of *ω* < 1 and/or *ω* = 1. Model M2a allows for an extra category of codon site compared to M1a which can evolve under positive selection (*ω* > 1). Model M8a assumes a discrete beta distribution for *ω*, which is constrained between 0 and 1 including a class with *ω* = 1. Model M8 allows the same distribution as M8a with an extra class of codons under positive selection with *ω* > 1.

Branch-site models allow *ω* to vary both among sites in the protein and across branches on the tree with the aim to detect positive selection affecting a few sites along particular branches (known as foreground branches). The branch-site A model was applied for branches leading to species with high or low leaf density; deciduous, evergreen or semi-evergreen species and species living in climates 1, 2, 3, 4, 5 and 6. When the number of species inhabiting a particular climate represented less than 15% of the total species analysed, then this climate was discarded for the branch site test. Model A1 allows 0 < *ω* < 1 and *ω* = 1 for all branches and also two additional classes of codons with fixed *ω* = 1 along pre-specified foreground branches while restricted as 0 < *ω* < 1 and *ω* = 1 on background branches. The alternative model A allows 0 < *ω* < 1 and *ω* = 1 for all branches and also two alternative classes of codons under positive selection with *ω* > 1 along pre-specified foreground branches while restricted to values of 0 < *ω* < 1 and *ω* = 1 on background branches.

We performed three LRTs to compare the nested site models M1a-M2a, M8-M8a and branch-site models A-A1. The LRTs values are calculated as twice the difference in the log-likelihood values of the models being compared, with the degrees of freedom being the difference in the number of parameters estimated in each of the models. LRT value can be approximated to a chi-square distribution. For the comparisons between M1a-M2a, M8-M8a and A-A1 the df was 2, 1 and 0.5, respectively.

### Coevolution analyses

CAPS software [86] was used to test for dependencies among amino acids on the RuBisCO structure. We used the Bayesian trees of 158 *Quercus* large and 174 Fagales as topology references for the analyses. CAPS compares the correlated variance of the evolutionary rates at two sites. This variance is corrected by the amount of divergence between the sequences compared using either the synonymous nucleotide substitutions or, alternatively, amino acid replacements as a relative measures of time. For each protein alignment, the corresponding BLOSUM matrix was applied depending on the average sequence identity. The significance of the results was evaluated by randomization of pairs of amino acid sites in the alignment, calculation of their correlation values, and comparison of the real values with the distribution of 10,000 randomly sampled values. An alpha value of 0.01 was applied to minimize the number of false positives. The level of substitutions per synonymous site weighted the correlated variability among amino acid sites in order to normalize parameters by the time of sequence divergence. The method detects phylogenetic-independent coevolution. We also conducted an analysis of the statistical support for each of the coevolving amino acid pair using a non-parametric bootstrap analysis. Briefly, for each pair exhibiting significant coevolution signatures we shuffled the sequences across the tree and we then re-ran CAPS on the new resulting alignment. We then identified coevolving pairs of amino acid sites and checked for the presence of the pair identified in the original non-random alignment. We repeated this procedure 1000 times and for each pairs of coevolving sites determined its frequency as the number of times it is detected in the 1000 replicates divided by 1000. A pair of coevolving sites was considered to be significantly represented in the bootstrap procedure when its frequency was equal or larger than 0.8.

### Decision tree model

Decision tree (DT) model analysis (“rpart” package in R v3.1.1) [87] was used to relate the proportion of amino acids present in all variable positions of the L-subunit of RuBisCO to species-specific traits (geographic area, climate, leaf habit and density), denoted as *external variables*.

For each variable position, the program builds a DT as follows. First, a question is found based on the analysis of all three external variables to split the species. Then, based on that question, the species are separated into two groups, in which the variability of that site is as low as possible. The analysis is repeated for each subgroup using all three external variables. The process continues until the lowest entropic error (xerror) for the entire DT is obtained [88]. The quality of the DT is categorized by its xerror as a function of the proportion of correct predictions and the complexity of the tree. The lower the xerror, the higher the relationship between the external variable and the variable site. Only DTs with xerror < 1 were selected. The program also calculates the importance of each external variable within the predictive model.

An advantage of DTs is that no statistical assumptions (about the independence, the distribution, the variance, etc.) are needed. The main limitation of DTs is to identify the optimal tree under certain criteria, so algorithms are employed to give an approximate solution.

## Results

### The *rbcL* variability

We obtained complete sequences of the *rbcL* gene (1428 nucleotides) for 158 *Quercus* species, 12 other Fagaceae species (6 *Fagus*, 3 *Castanea*, 1 *Castanopsis*, 2 *Lithocarpus*) and 4 Nothofagaceae species (4 *Nothofagus*). Within the Fagales dataset (Fagaceae and Nothofagaceae, all 174 species), 19 variable amino acid sites were observed, resulting in 30 haplotypes (i.e. group of species with identical L-subunit sequence) (S4 Table). Within *Quercus* (158 species), 9 variable sites defined the L-subunit and species were grouped into 21 haplotypes. In the two datasets, most of the species belonged to haplotype 1.

Complete sequences of the chloroplast *matK* gene and nuclear SSRs were obtained for 43 and 42 species, respectively (*Quercus* small dataset). The phylogenetic tree constructed for the *Quercus* small dataset with *rbcL*, *matK* and SSRs is well resolved with posterior probabilities > 50% (Fig 2). The tree topology was similar to that of Manos et al. (1999) [89] based on combined chloroplast DNA and nuclear internal transcribed spacers (ITS).

### Positive selection in *Quercus rbcL*

LRTs for positive selection (Table 1) indicated that the free-ratio model, that allows estimating *ω* for each of the branches of the tree, was significantly better than the models that do not allow for differences in *ω* values among tree branches (*p*-value = 0.0001). Models M2a and M8 both pointed to positive selection on *rbcL* in *Quercus* (small and large datasets) and Fagales. The three datasets exhibited positive selection at the amino acid sites 95, 219 and 328 (Table 1). In *Quercus*, asparagine (Asp) and serine (Ser) occurred at site 95, however threonine was found (Thr) in *Nothofagus antarctica* and *N*. *procera* (S4 Table). In the three groups, valine (Val) and leucine (Leu) occurred at site 219, and alanine (Ala) and Ser were found at position 328. In both the *Quercus* small and Fagales datasets, site 262 (Val or Ala) was positively selected. Sites 251 and 475 appeared as positively selected only in the *Quercus* large dataset. Isoleucine (Ile) and methionine (Met) occurred at residue 251, and Leu and Val occurred at residue 475. Site 145 in *Fagus*, *Lithocarpus* and *Nothofagus* was either a Val or Ala, although all *Quercus* species, *Castanopsis carlesi*, *Castanea pumila* and *Lithocarpus densiflorus* shared Ser.

**Table 1:** RuBisCO L-subunit sites subject to positive selection.

Dataset	N^a^	Site models				M2a *vs*. M1a test	Site model			M8 *vs*. M8a test
		M0	M2a				M8			
		*ω*^b^	*p*_2_^c^	*ω*_2_^d^	Selected sites^e^	*p*-value	*p*_1_^c^	*ω*^d^	Selected sites^e^	*p*-value
*Quercus* small	47	0.17	0.011	14.71	95**, 219**, 262*, 328**	0.000	0.011	14.71	95**, 219**, 262**, 328**	0.000
*Quercus* large	158	0.18	0.013	13.77	95**, 219**, 328**	0.000	0.017	11.32	95**, 219**, 251*, 328**, 475*	0.000
*Fagales*	174	0.16	0.009	17.05	95**, 219**, 262**, 328**	0.000	0.009	17.54	95**, 145*, 219**, 262**, 328**	0.000

Likelihood ratio tests (LRTs) were calculated between nested models of codon evolution M1a-M2a and M8-M8a.

^a^ Number of species.

^b^ *d*_N_*/d*_S_ ratio averaged across all branches and codons.

^c^ Proportion of codons in a class under positive selection.

^d^ *d*_N_*/d*_S_ ratio in a class under positive selection.

^e^ Sites marked with * and ** are under positive selection with posterior probability higher than 0.95 and 0.99, respectively.

In the Fagales dataset, LRT (Table 2) indicated that the branch-site model A (*ω*_2_ = estimated, in branches leading to deciduous or evergreen species or belonging to climate 5, see S1 and S2 Tables) was a significantly better fit to the data than the null model A1 (*ω*_2_ = 1, fixed) (*p*-value = 0.0001). However, no positively-selected sites were identified under the branch-site model A in either the *Quercus* small or large datasets. A total of five sites (95, 145, 251, 262 and 328) appeared as positively selected in Fagales, each exhibiting a posterior Bayesian probability greater than 0.90 (Table 2). In branches leading to evergreen species, Asp95Ser replacement was found at least two times (*Q*. *germana*, *C*. *carlesii*) (Table 3). In branches leading to deciduous species, i) Ile251Met replacement was found at least six times (*Q*. *aliena*, *Q*. *fabri*, *Q*. *griffithii*, *Q*. *muehlenbergii*, *Q*. *serrrata* var. *brevipetiolata* and *Q*. *wutaishanica*); ii) Ala262Val replacement occurred on one branch leading to *C*. *sativa*; iii) Ala328Ser replacement occurred in branches leading to *Q*. *eugeniifolia* and *Q*. *seemani*, although Ser328Ala replacement occurred instead in branches leading to *N*. *procera* and *N*. *antarctica*. In branches leading to species in climate 5, i) Ser145Ala replacement took place in at least six times (*F*. *crenata*, *F*. *japonica*, *F*. *lucida*, *F*. *sylvatica*, *N*. *procera*, *and L*. *hancei*), while Ser145Val replacement occurred at least three times in branches leading to *N*. *menziesii*, *N*. *moorei* and *N*. *antarctica*, ii) Ala262Val replacement occurred in the branch leading to *C*. *sativa*; iii) Ala328Ser replacement was found in branches leading to *Q*. *costaricensis*, while Ser328Ala replacement occurred in branches leading to *C*. *sativa*, *C*. *mollissima*. *N*. *procera*, *N*. *menziesii*, *N*. *moorei* and *N*. *antarctica*.

**Table 2:** Results of branch site model A in Fagales dataset (174 species).

Parameters^a^	N^b^	Branch site model			MA *vs*. MA1 test
		MA			
		*p*_2_^c^	*ω*_2_^d^	Selected sites^e^	*p*-value
High leaf density (>900 kg m^−3^)	48	0.002	94.3	328**	1
Low leaf density (<600 kg m^−3^)	67	0.000	999.0	-	1
Evergreen	75	0.00023	999.0	95*** 262***	0.000
Semi-evergreen	17	0.000	4.47	-	1
Deciduous	82	0.005	39.7	251**, 262***, 328***	0.000
Climate 1	15	0.028	999.0	-	0.000
Climate 3	41	0.000	999.0	-	1
Climate 4	35	0.000	999.0	262**	1
Climate 5	69	0.004	22.03	145*, 262***, 328***	0.000

Likelihood ratio tests (LRTs) were performed to compare the null model A1 (that assumes the same selective pressure along all branches of a phylogeny) with the nested model A (that aims to detect positive selection along particular lineages, forward branches).

^a^ According to information in S1 Table.

^b^ Number of species labelled as forward branches.

^c^ Proportion of codons in a class under positive selection.

^d^ *d*_N_*/d*_S_ ratio in a class under positive selection.

^e^ Sites marked with *, ** and *** are under positive selection with posterior probability higher than 0.90, 0.95 and 0.99, respectively.

**Table 3:** RuBisCO L-subunit amino acid replacements in Fagales (174 species) identified under positive selection by the Bayes Empirical Bayes (BEB) analysis implemented in the PAML package [83, 90] along branches leading to species with particular leaf or habitat trait.

Species	Amino acid changes	Location of residue	Interactions^b^
Branches leading to evergreen species			
95	Asn **→** Ser	Loop between βC-βD strand	RuBisCO activase
Branches leading to deciduous species			
251	Ile **→** Met	Helix α3	
262	Ala **→** Val	Loop 3	S-subunit
328	Ala **→** Ser/ Ser **→** Ala	Loop 6	Active site
Branches leading to species inhabiting climate 5			
145	Ser **→** Ala/Val	Helix αD	Hidrophobic core between N-terminal and C-terminal domains
262	Ala **→** Val	Loop 3	S-subunit
328	Ala **→** Ser/ Ser **→** Ala	Loop 6	Active site

### Analysis of dependent evolution among amino acid sites in *rbcL*

**Fig 4:**
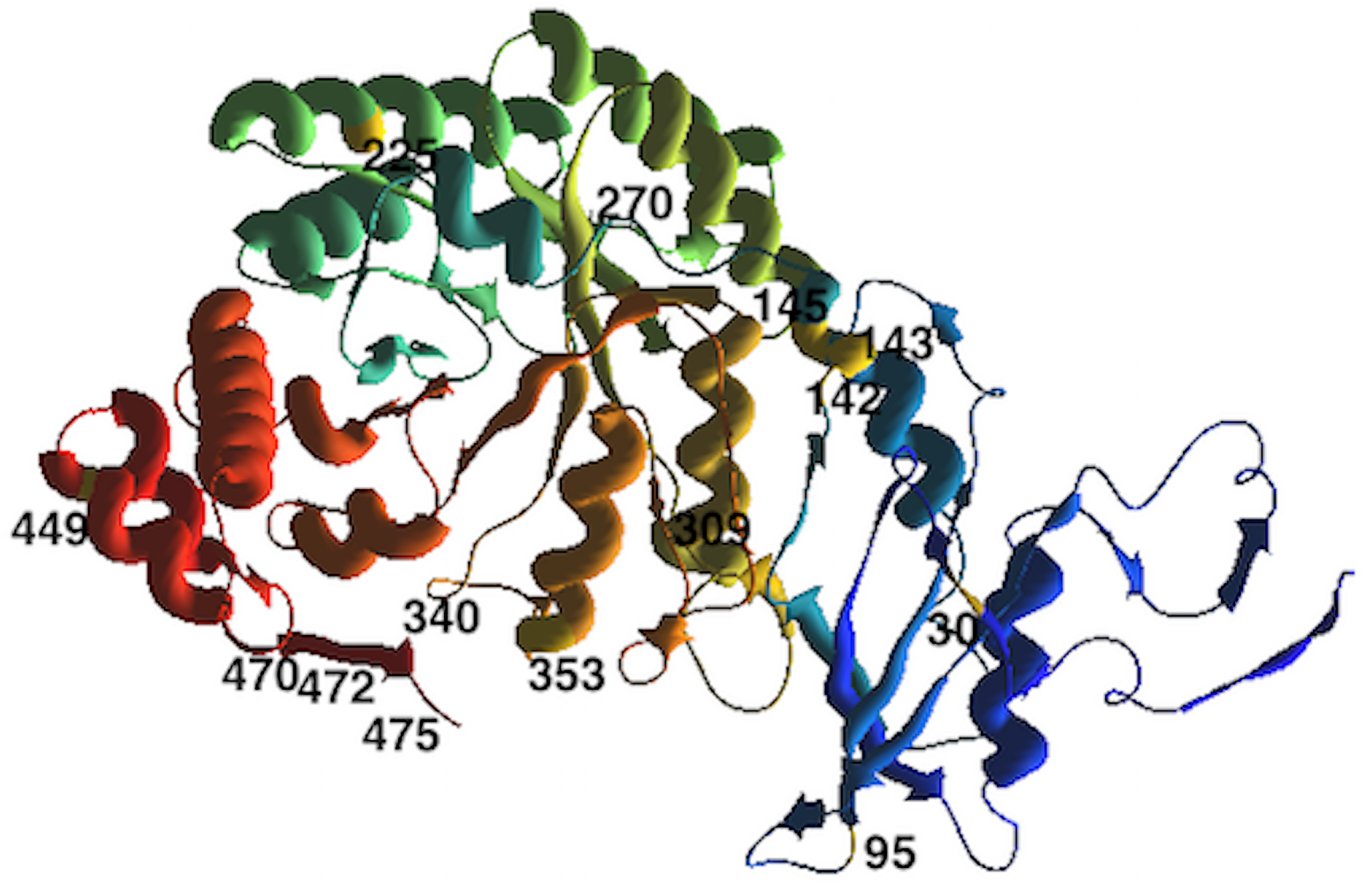
Coevolving sites in the RuBisCO L-subunit of Fagales dataset. Location of amino acids implicated in co-evolutionary dependencies.

Analysis of coevolution in *rbcL* identified 29 pairs of coevolving amino acids in Fagales dataset with a total of 14 non-redundant amino acids involved (Fig 4 and S5 Table). The largest group of coevolution was composed of sites with only one interaction, and included residues 95, 143, 225, 449, 472 and 475. The coevolving pairs with the highest correlations (1.0) (as a measure of coevolution) were 30–449, 270–340, 270–353, 270–470, 340–353, 340–470, 353–470 and 472–475. In the *Quercus* large dataset, no amino acid were detected as coevolving.

### Decision tree model

**Fig 5:**
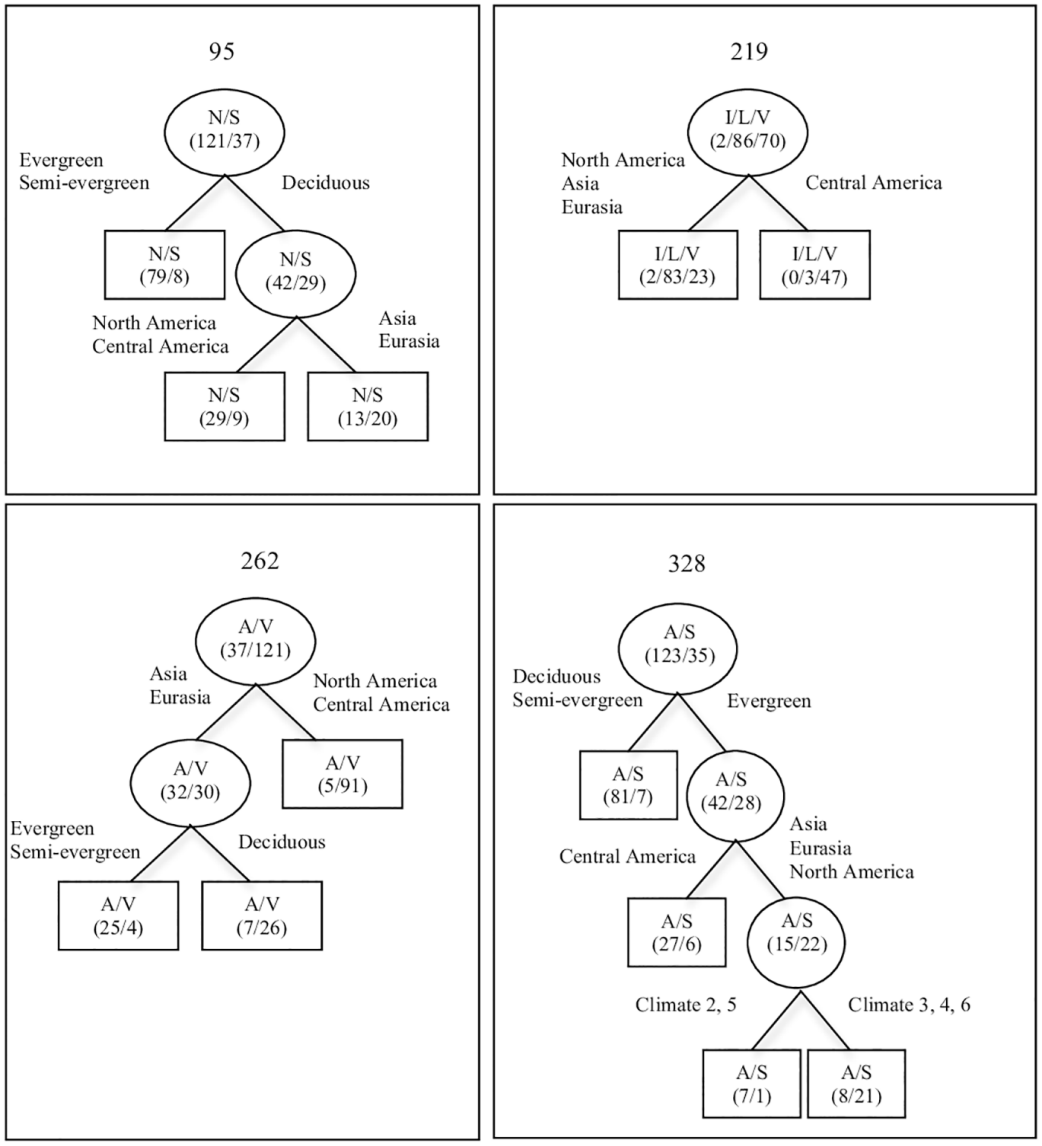
Decision trees (DT) resolved for each RuBisCO L-subunit variable site in *Quercus* large dataset (158 species) (see S1 Table for details on the external variables: Geographic distribution, climate and leaf habit and density). Numbers above each tree correspond to the RuBisCO L-subunit variable site according to the spinach sequence (AJ400848.1). First level presents the proportion of amino acids in each variable site (brackets). The external variable that allows the best separation of species is shown over the line. The second level presents the distribution of amino acids (in brackets) after the first split. Subsequent divisions are performed until the lowest xerror for the entire DT is obtained (symbolized as squares). Taking as an example the RuBisCO L-subunit variable site 95, the first level shows the separation of the 158 species between those that present N (121) and those that present S (37). Over the line, leaf habit is indicated as the external variable that gives the best split among the four external variables, with evergreen and semi-evergreen species having a proportion of N/S of 79/8. On the other hand, deciduous species present a proportion of N/S of 42/29. The latter group is further split using geographic area as the best external variable into a group of species from North and Central America having a N/S proportion of 29/9, and a group of species from Eurasia and Asia having a proportion of 13/20. The relative importance of each external variable is shown in Table 4.

In the *Quercus* large dataset (158 species), the DT model pointed to a link between the external variables (geographic distribution, climate and leaf habit and density) and the RuBisCO L-subunit variable sites 95, 219, 262 and 328 (Table 4, Fig 5). The xerrors calculated for each variable site were 0.89, 0.39, 0.44 and 0.59, respectively. According to the xerror, the sites that were best explained by the external variables were 219 and 262 followed by 328 and 95. The leaf habit (evergreen, semi-evergreen and deciduous) was the external variable that best explained variability at site 95, followed by the geographic area, climate and leaf density (Table 4). The most important variable for sites 219 and 262 was geographic area, followed by climate and leaf density for site 219 and leaf habit, leaf density and climate for site 262. Site 328 was best explained by climate (2 or 5), followed by geographic area, leaf habit, and density (Table 4, Fig 5).

**Table 4:** Variable sites resolved with the DT model for the *Quercus* large dataset (158 species).

Variable site	xerror	Relative importance of external variables			
		Geographic area	Climate	Leaf habit	Leaf density
95	0.89	25	20	35	20
219	0.39	66	26	n.a.	8
262	0.44	41	14	27	18
328	0.59	28	32	27	13

The xerror corresponding to the best DT found for each variable site, and relative importance (%) of the external variables (geographic area, climate and leaf habit and density) calculated for each resolved sites are shown. The lower the xerror, the higher the relationship between the external variables and the variable site. The external variable with the higher relative importance is the most important external variable explaining the variability in the site. n.a. denotes not selected external variable.

## Discussion

### Both phylogenetic analyses by maximum likelihood and decision tree analyses highlighted the same amino acid substitutions

Methodologically different approaches have been used to study molecular adaptation of RuBisCO to particular ecological conditions in oak trees (*Quercus*). Phylogenetic analysis by maximum likelihood (PAML) [90] is a standard method to identify positive selection at the molecular level. In the present study, six RuBisCO L-subunit sites (95, 219, 251, 262, 328 and 475) were identified by models M2a and M8 to have evolved under positive selection in *Quercus* large and small datasets (Table 1), all of which were previously reported in other groups of plants [41, 42, 43, 44]. For the same *Quercus* datasets, we compared the results of another method, the DT model. DT linked RuBisCO L-subunit sites 95, 219, 262 and 328 to distribution, climate, leaf habit and density (Table 4). All four variable sites resolved by the DT model were positively selected according to maximum likelihood analyses (Tables 1 and 4). The analytically simple DT method combined with PAML provided evidence of a link between amino acids replacements in RuBisCO and specific phenotypes [84, 85]. The combination of both methods constitutes a powerful tool to identify causal links between genetic variants and adaptation of the L-subunit of RuBisCO.

According to the DT model analysis, replacement at site 95 was linked to the leaf habit as the most important external variable (Table 4). Since site 95 evolved under positive selection (Table 1), and evergreen and deciduous species typically display different mesophyll conductance (*g*_m_) influencing the CO_2_ concentration at the site of carboxylation [63], this result provides support to the idea that CO_2_ availability shapes RuBisCO evolution [14, 91].

The six RuBisCO sites under positive selection in *Quercus* large and small datasets (Table 1) were located in functionally important subunit interfaces within the RuBisCO complex (95, 219, 251, 262, 328 and 475). Site 95 was hypothesized to be involved in interactions between RuBisCO and RuBisCO activase [92, 93]. Sites 219 and 262 were reported to be involved in interactions between large and small subunits [94]. Site 262 is located in loop 3 in a hydrophobic core in the C-terminal α-β barrel domain and could influence holoenzyme thermal stability and catalysis [95]. Site 251 seems to be involved in dimer-dimer interactions within the large subunits [96] and sites 328 and 475 are located close to the active site and in the C-terminus, respectively [95, 97].

### Evidence for CO_2_ as a major factor driving RuBisCO evolution in Fagales

In Fagales, species with evergreen leaves had RuBisCO residues 95 and 262 under positive selection, species with deciduous leaves had residues 251, 262 and 328 under positive selection, and species inhabiting climate number 5 had residues 145, 262 and 328 under positive selection (Table 2). In species with evergreen leaves, amino acid replacements in position 95 (Table 3) may be linked to an increased affinity of RuBisCO for CO_2_ (i.e., low values of the RuBisCO Michaelis-Menten constant, *K*_c_). The work by Galmés et al. [18] linked the Asp95Ser replacement to high affinity for CO_2_ (low *K*_c_). The dataset of evergreen Fagales had an average LMA of 117.5 ± 0.4 g m^-2^. This high LMA, and specifically high leaf density, could have been associated with high resistance to CO_2_ internal diffusion [63, 97]. Moreover, species with high LMA also tend to present lower values of stomatal conductance [98]. Hence, RuBisCO of evergreen Fagales probably works at relatively low CO_2_ partial pressures. Taken together, these results suggest that amino acid replacements at position 95 in evergreen Fagales may lead to RuBisCO with increased affinity for CO_2_. Unfortunately, our attempts to extract active RuBisCO in different Fagales failed due to the high content of polyphenols and other secondary metabolic compounds. Future efforts will demand testing different extraction buffers to obtain sufficient active enzyme and determine key RuBisCO kinetic parameters. In species with deciduous leaves or inhabiting areas lacking a dry season (climate number 5), replacements at site 328 (Table 3) could be related with decreased RuBisCO CO_2_ affinity (i.e., high values of *K*_c_). Galmés et al. [15] reported low specificity factor (*S*_c/o_) and high maximum carboxylase turnover rate (*k*_cat_^c^) and *K*_c_ in *Limonium* species having serine at the site 328. Those kinetic values are associated with an increase in the chloroplast concentration of CO_2_. Within Fagales, species with deciduous leaves or belonging to temperate climate (climate number 5) had average LMA value of 87.6 ± 0.2 and 95.4 ± 0.4 g m^-2^, respectively. Low LMA and low leaf density, together with the absence of a dry season, may favour high CO_2_ concentration in the stroma of the chloroplast, via indirect effects on leaf conductance to CO_2_ [98]. RuBisCO in deciduous species or inhabiting climate number 5 could have adapted towards a higher *k*_cat_^c^ and lower CO_2_ affinity (high *K*_c_) and *S*_c/o_, although this could be confirmed with future kinetic analyses.

In total, twenty-nine residue pairs in Fagales RuBisCO L-subunit were linked through intramolecular coevolution, representing c.a. 3% of L-subunit residues (14 out of 476) (Fig 4 and S5 Table). Many of the coevolving residues detected in the present study were already reported as coevolving in previous studies including different land plant lineages [47, 56]. Our results showed that both positive selection and coevolution affect some sites. For example, site 95 was positively selected within evergreen species (Tables 1 and 2) and appeared as coevolving with site 309 (S5 Table). By contrast, site 145 was positively selected within species of climate number 5 (Tables 1 and 2) and appeared as coevolving with seven sites (30, 142, 143, 270, 340, 353, 470) (S5 Table). Three of the coevolving pairs, 30–340, 142–470 and 270–309 coevolve in terms of their physic-chemical properties, including molecular weight and hydrophobicity (S5 Table). Coevolving sites may be located within structurally and/or functionally important positions. For example, Kellogg and Juliano (1997) [99] reported the importance of sites 142 and 145 for dimer-dimer association, and sites 225 and 449 could be important for the interaction between large and small subunits [100]. Knowledge of co-evolution networks operating in RuBisCO L-subunit of Fagales provides useful information on target substitutions to improve the catalytic performance of RuBisCO.

### Concluding remarks

Based on our research, it is reasonable to postulate that finely tuned biochemical properties of *Quercus* RuBisCOs have evolved as a result of environmental pressures. Such evolution is manifested by positively selected amino acid replacements within the large subunits of *Quercus* RuBisCO, which are likely related to different physiological and environmental traits. These changes could have fine-tuned RuBisCO catalytic efficiency and may have facilitated *Quercus* adaptive radiation into diverse ecological niches. The DT model and phylogenetic analysis by maximum likelihood identified the same amino acid replacements associated with ecological adaptation and positive selection.

## Supporting information

S1 Table Studied species from Fagaceae and Nothofagaceae families from the order Fagales. The genus, subgenus and section are indicated along with information on the species distribution, climate, and leaf habit and density. Data on the geographic distribution and leaf habit were obtained from Govaerts et al. (1998) [64] and publicly available databases [65, 66, 67, 68]. The climate types were obtained by overlapping the species geographical distribution and the Köppen-Geiger world map of climate classification [69]. Fifteen different Köppen-Geiger types of climates were grouped into six: 1 = tropical, 2 = arid steppe, 3 = temperate with dry winter and hot or warm summer, 4 = temperate with dry summer and hot or warm summer, 5 = temperate or cold without dry season and hot or warm summer, 6 = cold with dry summer and hot or warm summer. The species leaf density was calculated from leaf thickness and leaf mass area (LMA) measurements. The three columns on the right correspond with [1] classification.
(PDF)

S2 Table Köppen-Geiger climate types assigned to each of the 174 species (S1 Table) based on the geographic area. To simplify the analysis, 15 different Köppen-Geiger types of climates were grouped into six: 1) tropical (including climates Af, Am and Aw according to Köppen-Geiger classification); 2) arid steppe (Bsh, Bsk); 3) temperate with dry winter and hot or warm summer (Cwa, Cwb); 4) temperate with dry summer and hot or warm summer (Csa, Csb); 5) temperate or cold without dry season and hot or warm summer (Cfa, Cfb, Dfa, Dfb), and 6) cold with dry summer and hot or warm summer (Dsa, Dsb).
(PDF)

S3 Table Fagaceae and Nothofagaceae GenBank accession numbers for *rbcL* and *matk* genes. (PDF)

S4 Table RuBisCO L-subunit 19 variable sites identified in 174 Fagales species, grouped in 30 haplotypes (i.e., groups of species with identical L-subunit sequence). Variable sites identified when *Quercus* large dataset (158 species) were analyzed separately are marked in grey (9 variable sites and 21 haplotypes). Species marked with an asterisk were used to construct the *Quercus* small dataset (45 species) phylogeny based on *rbcL*, *matK* and SSRs.
(PDF)

S5 Table Coevolution pairs within the L-subunit of RuBisCO. Significant correlation coefficients are shown (*p* < 0.001). Mean D1 and Mean D2 values correspond to the mean distance calculated for each coevolving position based on BLOSUM as calculated in the method of Fares and Travers (2006) [1]. The bootstraps are based on 100 resampling, the confidence level associated with the pair that coevolves (values greater than 75% are significant after a non-parametric resampling over the tree). Atomic distances were calculated from 3D crystal structure wherever available by measuring the average Euclidean distance between atoms of two amino acids (Å). Atomic distances are not used here as evidence of coevolution but rather as additional supporting information in the identification of functional and structural coevolution. A test for variability in hydrophobicity and molecular weight has been also conducted giving as a result the pair 30–340, 142–470 and 270–309. n.a. not available.
(PDF)

## References

[1] Whitney SM, Houtz RL, Alonso H. Advancing our understanding and capacity to engineer nature’s CO_2_-sequestering enzyme, Rubisco. Plant Physiol. 2011; 155:27–35. doi: 10.1104/pp.110.164814 20974895 10.1104/pp.110.164814 PMC3075749

[2] Ellis RJ. The most abundant protein on earth. Trends Biochem Sci. 1979; 4:241–244.

[3] Berry J, Bjorkman O. Photosynthetic response and adaptation to temperature in higher plants. Ann Rev Plant Physio. 1980; 31:491–543.

[4] Ehleringer J, Mooney HA. Productivity of desert and Mediterranean-climate plants In: Lange OL, Nobel PS, Osmond CB, Ziegler H, editors. Physiological plant ecology IV. Springer Berlin Heidelberg, 1983; pp 205–231.

[5] Yamori W, Takahashi S, Makino A, Price GD, Badger MR, von Caemmerer S. The roles of ATP synthase and the cytochrome b6/f complexes in limiting chloroplast electron transport and determining photosynthetic capacity. Plant Physiol. 2011; 155:956–962. doi: 10.1104/pp.110.168435 21177473 10.1104/pp.110.168435 PMC3032479

[6] Flexas J, Diaz-Espejo A, Gago J, Gallé A, Galmés J, Gulías J, et al Photosynthetic limitations in Mediterranean plants: a review. Environ Exp Bot. 2014; 103:12–23.

[7] Hozain MDI, Salvucci ME, Fokar M, Holaday AS. The differential response of photosynthesis to high temperature for a boreal and temperate Populus species relates to differences in Rubisco activation and Rubisco activase properties. Tree Physiol 2010; 30:32 doi: 10.1093/treephys/tpp091 19864261 10.1093/treephys/tpp091

[8] Galmés J, Ribas-Carbó M, Medrano H, Flexas J. Rubisco activity in Mediterranean species is regulated by the chloroplastic CO_2_ concentration under water stress. J Exp Bot. 2011; 62:653–665. doi: 10.1093/jxb/erq303 21115663 10.1093/jxb/erq303 PMC3003812

[9] Delgado E, Medrano H, Keys AJ, Parry MAJ. Species variation in Rubisco specificity factor. J Exp Bot. 1995; 46:1775–1777.

[10] Raven JA. Land plant biochemistry. Philos T R Soc B. 2000; 355:833–846. 10.1098/rstb.2000.0618 PMC1692786 10905612

[11] Sage RF. Variation in the kcat of Rubisco in C_3_ and C_4_ plants and some implications for photosynthetic performance at high and low temperature. J Exp Bot. 2002; 53:609–620. 11886880 10.1093/jexbot/53.369.609

[12] Galmés J, Flexas J, Keys AJ, Cifre J, Mitchell RAC, Madgwick PJ, et al Rubisco specificity factor tends to be larger in plant species from drier habitats and in species with persistent leaves. Plant Cell Environ. 2005; 28:571–579.

[13] Tcherkez GG, Farquhar GD, Andrews TJ. Despite slow catalysis and confused substrate specificity, all ribulose bisphosphate carboxylases may be nearly perfectly optimized. P Natl Acad Sci. 2006; 103:7246–7251. 10.1073/pnas.0600605103 PMC1464328 16641091

[14] Savir Y, Noorb E, Milob R, Tlustya T. Cross-species analysis traces adaptation of Rubisco toward optimality in a low-dimensional landscape. P Natl Acad Sci USA. 2010; 107:3475–3480. 10.1073/pnas.0911663107 PMC2840432 20142476

[15] Galmes J, Andralojc PJ, Kapralov MV, Flexas J, Keys AJ, Molins A, et al Environmentally driven evolution of Rubisco and improved photosynthesis and growth within the C_3_ genus Limonium (Plumbaginaceae). New Phytol. 2014; 203:989–999. doi: 10.1111/nph.12858 24861241 10.1111/nph.12858

[16] Galmés J, Conesa MÀ, Díaz-Espejo A, Mir A, Perdomo JA, Niinemets Ü, et al Rubisco catalytic properties optimized for present and future climatic conditions. Plant Sci. 2014; 226:61–70. doi: 10.1016/j.plantsci.2014.01.008 25113451 10.1016/j.plantsci.2014.01.008

[17] Zhu XG, Portis AR, Long SP. Would transformation of C_3_ crop plants with foreign Rubisco increase productivity? A computational analysis extrapolating from kinetic properties to canopy photosynthesis. Plant Cell Environ. 2004; 27:155–165.

[18] Galmes J, Kapralov MV, Andralojc P, Conesa MÀ, Keys AJ, Parry MA, et al Expanding knowledge of the Rubisco kinetics variability in plant species: environmental and evolutionary trends. Plant Cell Environ. 2014; 37:1989–2001. doi: 10.1111/pce.12335 24689692 10.1111/pce.12335

[19] Yeoh HH, Badger MR, Watson L. Variations in Km (CO_2_) of ribulose-1,5-bisphosphate carboxylase among grasses. Plant Physiol. 1980; 66:1110–1112. 16661586 10.1104/pp.66.6.1110 PMC440799

[20] Yeoh HH, Badger MR, Watson L. Variations in kinetic properties of ribulose-1,5-bisphosphate carboxylase among plants. Plant Physiol. 1981; 67:1151–1155. 16661826 10.1104/pp.67.6.1151 PMC425851

[21] Seemann JR, Badger MR, Berry JA. Variations in the specific activity of ribulose-1,5-bisphosphate carboxylase between species utilizing differing photosynthetic pathways. Plant Physiol. 1984; 74:791–794. 16663511 10.1104/pp.74.4.791 PMC1066769

[22] Ghannoum O, Evans JR, Chow WS, Andrews TJ, Conroy JP, Von Caemmerer S. Faster Rubisco is the key to superior nitrogen-use efficiency in NADP-malic enzyme relative to NAD-malic enzyme C_4_ grasses. Plant Physiol. 2005; 137:638–650. doi: 10.1104/pp.104.054759 15665246 10.1104/pp.104.054759 PMC1065364

[23] Kubien DS, Whitney SM, Moore PV, Jesson LK. The biochemistry of Rubisco in Flaveria. J Exp Bot. 2008; 59:1767–1777. doi: 10.1093/jxb/erm283 18227079 10.1093/jxb/erm283

[24] Carmo-Silva AE, Keys AJ, Andralojc PJ, Powers SJ, Arrabaça MC, Parry MA. Rubisco activities, properties, and regulation in three different C_4_ grasses under drought. J Exp Bot. 2010; erq071. 10.1093/jxb/erq071 PMC2877893 20363871

[25] Brooks A, Farquhar GD. Effect of temperature on the CO_2_/O_2_ specificity of ribulose-1, 5-bisphosphate carboxylase/oxygenase and the rate of respiration in the light. Planta. 1985; 165:397–406. doi: 10.1007/BF00392238 24241146 10.1007/BF00392238

[26] Uemura K, Miyachi S, Yokota A. Ribulose-1,5-bisphosphate carboxylase/oxygenase from thermophilic red algae with a strong specificity for CO_2_ fixation. Biochem Bioph Res Co. 1997; 233:568–571. 10.1006/bbrc.1997.6497 9144578

[27] Andersson I, Backlund A. Structure and function of Rubisco. Plant Physiol Bioch. 2008; 46:275–291. 10.1016/j.plaphy.2008.01.001 18294858

[28] Spreitzer RJ, Salvucci ME. Rubisco: structure, regulatory interactions, and possibilities for a better enzyme. Annu Rev Plant Biol. 2002; 53:449–475. doi: 10.1146/annurev.arplant.53.100301.135233 12221984 10.1146/annurev.arplant.53.100301.135233

[29] Genkov T, Spreitzer RJ. Highly conserved small subunit residues influence rubisco large subunit catalysis. J Biol Chem, 2009; 284:30105–30112. doi: 10.1074/jbc.M109.044081 19734149 10.1074/jbc.M109.044081 PMC2781565

[30] Ishikawa C, Hatanaka T, Misoo S, Miyake C, Fukayama H. Functional incorporation of sorghum small subunit increases the catalytic turnover rate of Rubisco in transgenic rice. Plant Physiol. 2011; 156:1603–1611. doi: 10.1104/pp.111.177030 21562335 10.1104/pp.111.177030 PMC3135941

[31] Morita K, Hatanaka T, Misoo S, Fukayama H. Unusual small subunit that is not expressed in photosynthetic cells alters the catalytic properties of Rubisco in rice. Plant Physiol. 2014; 164:69–79. doi: 10.1104/pp.113.228015 24254313 10.1104/pp.113.228015 PMC3875826

[32] Kanevski I, Maliga P, Rhoades DF, Gutteridge S. Plastome engineering of ribulose-1,5- bisphosphate carboxylase/oxygenase in tobacco to form a sunflower large subunit and tobacco small subunit hybrid. Plant Physiol. 1999; 119:133–141. 9880354 10.1104/pp.119.1.133 PMC32212

[33] Sharwood RE, von Caemmerer S, Maliga P, Whitney SM. The catalytic properties of hybrid Rubisco comprising tobacco small and sunflower large subunits mirror the kinetically equivalent source Rubiscos and can support tobacco growth. Plant Physiol. 2008; 146:83–96. doi: 10.1104/pp.107.109058 17993544 10.1104/pp.107.109058 PMC2230571

[34] Whitney SM, Sharwood RE, Orr D, White SJ, Alonso H, Galmés J. Isoleucine 309 acts as a C_4_ catalytic switch that increases ribulose-1,5-bisphosphate carboxylase/oxygenase (rubisco) carboxylation rate in Flaveria. P Natl Acad Sci. 2011; 108:14688–14693. 10.1073/pnas.1109503108 PMC3167554 21849620

[35] Zhang XH, Webb J, Huang YH, Lin L, Tang RS, Liu A. Hybrid Rubisco of tomato large subunits and tobacco small subunits is functional in tobacco plants. Plant Sci. 2011; 180:480–488. doi: 10.1016/j.plantsci.2010.11.001 21421395 10.1016/j.plantsci.2010.11.001

[36] Galmés J, Aranjuelo I, Medrano H, Flexas J. Variation in Rubisco content and activity under variable climatic factor. Photosynth Res. 2013; 117:73–90. doi: 10.1007/s11120-013-9861-y 23748840 10.1007/s11120-013-9861-y

[37] Occhialini A, Lin MT, Andralojc PJ, Hanson MR, Parry MA. Transgenic tobacco plants with improved cyanobacterial Rubisco expression but no extra assembly factors grow at near wild-type rates if provided with elevated CO_2_. Plant J. 2016; 85:148–160. doi: 10.1111/tpj.13098 26662726 10.1111/tpj.13098 PMC4718753

[38] Hermida-Carrera C, Kapralov MV, Galmés J. Rubisco catalytic properties and temperature response in crops. Plant Physiol. 2016; 171:2549–2561. doi: 10.1104/pp.16.01846 27329223 10.1104/pp.16.01846 PMC4972260

[39] Orr DJ, Alcântara A, Kapralov MV, Andralojc PJ, Carmo-Silva E, Parry MA. Surveying Rubisco diversity and temperature response to improve crop photosynthetic efficiency. Plant Physiol. 2016; 172:707–717. doi: 10.1104/pp.16.00750 27342312 10.1104/pp.16.00750 PMC5047088

[40] Sharwood RE, Ghannoum O, Kapralov MV, Gunn LH, Whitney S. Temperature responses of Rubisco from Paniceae grasses provide opportunities for improving C_3_ photosynthesis. Nat Plants. 2016 doi: 10.1038/nplants.2016.186 27892943 10.1038/nplants.2016.186

[41] Kapralov MV, Filatov DA. Molecular adaptation during adaptive radiation in the Hawaiian endemic genus Schiedea. PLoS One. 2006; 1:e8 doi: 10.1371/journal.pone.0000008 17183712 10.1371/journal.pone.0000008 PMC1762304

[42] Kapralov MV, Filatov DA. Widespread positive selection in the photosynthetic Rubisco enzyme. BMC evolutionary biology. 2007; 7:73 doi: 10.1186/1471-2148-7-73 17498284 10.1186/1471-2148-7-73 PMC1884142

[43] Christin PA, Salamin N, Muasya AM, Roalson EH, Russier F, Besnard G. Evolutionary switch and genetic convergence on *rbcL* following the evolution of C_4_ photosynthesis. Mol Biol Evol. 2008; 25:2361–2368. doi: 10.1093/molbev/msn178 18695049 10.1093/molbev/msn178

[44] Iida S, Miyagi A, Aoki S, Ito M, Kadono Y, Kosuge K. Molecular adaptation of rbcL in the heterophyllous aquatic plant Potamogeton. PLoS One. 2009; 4:e4633 doi: 10.1371/journal.pone.0004633 19247501 10.1371/journal.pone.0004633 PMC2646136

[45] Kato S, Misawa K, Takahashi F, Sakayama H, Sano S, Kosuge K, et al Aquatic plant speciation affected by diversifying selection of organelle DNA regions. J phycol. 2011; 47:999–1008. doi: 10.1111/j.1529-8817.2011.01037.x 27020181 10.1111/j.1529-8817.2011.01037.x

[46] Miwa H, Odrzykoski IJ, Matsui A, Hasegawa M, Akiyama H, Jia Y, Murakami N. Adaptive evolution of *rbc*L in Conocephalum (Hepaticae, bryophytes). Gene. 2009; 441:169–175. doi: 10.1016/j.gene.2008.11.020 19100313 10.1016/j.gene.2008.11.020

[47] Sen L, Fares MA, Liang B, Gao L, Wang B, Wang T, Su YJ. Molecular evolution of *rbcL* in three gymnosperm families: identifying adaptive and coevolutionary patterns. Biol Direct. 2011; 6:29 doi: 10.1186/1745-6150-6-29 21639885 10.1186/1745-6150-6-29 PMC3129321

[48] Kapralov MV, Kubien DS, Andersson I, Filatov DA. Changes in Rubisco kinetics during the evolution of C_4_ photosynthesis in Flaveria (Asteraceae) are associated with positive selection on genes encoding the enzyme. Mol Biol Evol. 2011; 28:1491–1503. doi: 10.1093/molbev/msq335 21172830 10.1093/molbev/msq335

[49] Kapralov MV, Smith JAC, Filatov DA. Rubisco evolution in C_4_ eudicots: an analysis of Amaranthaceae sensu lato. PLoS One. 2012; 7:e52974 doi: 10.1371/journal.pone.0052974 23285238 10.1371/journal.pone.0052974 PMC3527620

[50] Young JN, Rickaby REM, Kapralov MV, Filatov DA. Adaptive signals in algal Rubisco reveal a history of ancient atmospheric carbon dioxide. Philos T R Soc B. 2012; 367:483–492. 10.1098/rstb.2011.0145 PMC3248704 22232761

[51] Weinreich DM, Watson RA, Chao L. Perspective: sign epistasis and genetic constraint on evolutionary trajectories. Evolution. 2005 59:1165–1174. 16050094

[52] Raines CA. Transgenic approaches to manipulate the environmental responses of the C_3_ carbon fixation cycle. Plant Cell Environ. 2006; 29:331–339. 17080589 10.1111/j.1365-3040.2005.01488.x

[53] Dutheil J. Evolution and Structure of Biomolecules. Evolution. 2008; (1/23).

[54] Jiang X, Fares MA. Identifying coevolutionary patterns in human leukocyte antigen (Hla) molecules. Evolution. 2010; 64:1429–1445. doi: 10.1111/j.1558-5646.2009.00903.x 19930454 10.1111/j.1558-5646.2009.00903.x

[55] Codoñer FM, Fares MA. Why should we care about molecular coevolution? Evol bioinform. 2008; 4:29. PMC2614197 19204805

[56] Wang M, Kapralov MV, Anisimova M. Coevolution of amino acid residues in the key photosynthetic enzyme Rubisco. BMC Evolutionary Biology. 2011; 11:266 doi: 10.1186/1471-2148-11-266 21942934 10.1186/1471-2148-11-266 PMC3190394

[57] Schwabacher M, Aguilar R, Figueroa F. Using decision trees to detect and isolate simulated leaks in the J-2X rocket engine. IEEE Aeros Conf. 2009; pp 1–7.

[58] Simard M, Saatchi SS, De Grandi G. The use of decision tree and multiscale texture for classification of JERS-1 SAR data over tropical forest. IEEE T Geosci Remote. 2000; 38:2310–2321.

[59] Yang CC, Prasher SO, Enright P, Madramootoo C, Burgess M, Goel PK, Callum I. Application of decision tree technology for image classification using remote sensing data. Agr Syst. 2003; 76:1101–1117.

[60] Soleimanian F, Mohammadi P, Hakimi P. Application of Decision Tree Algorithm for Data Mining in Healthcare Operations: A Case Study. Int J Comput Appl. 2012; 52:21–26.

[61] James G, Witten D, Hastie T, Tibshirani R. An Introduction to Statistical Learning. 2015 New York: Springer p. 315.

[62] Corcuera L, Camarero JJ, Gil-Pelegrín E. Functional groups in Quercus species derived from the analysis of pressure-volume curves. Trees-Struct Funct. 2002; 16:465–472.

[63] Flexas J, Ribas-Carbó M, Diaz-Espejo A, Galmes J, Medrano H. Mesophyll conductance to CO_2_: current knowledge and future prospects. Plant Cell Environ. 2008; 31:602–621. doi: 10.1111/j.1365-3040.2007.01757.x 17996013 10.1111/j.1365-3040.2007.01757.x

[64] Govaerts R, Frodin DG. World checklist and bibliography of Fagales (Betulaceae, Corylaceae, Fagaceae and Ticodendraceae). 1st ed Kew, UK: The Royal Botanic Gardens; 1998.

[65] http://www.efloras.org/.

[66] http://esp.cr.usgs.gov/data/little/.

[67] http://www.kew.org/.

[68] http://www.luomus.fi/en/atlas-florae-europaeae-afe-distribution-vascular-plants-europe.

[69] Peel MC, Finlayson BL, McMahon TA. Updated world map of the Köppen-Geiger climate classification. Hydrol Earth Syst Sc. 2007; 4:439–473.

[70] Sancho-Knapik D, Álvarez-Arenas TG, Peguero-Pina JJ, Fernández V, Gil-Pelegrín E. Relationship between ultrasonic properties and structural changes in the mesophyll during leaf dehydration. J Exp Bot. 2011; 62:3637–3645. doi: 10.1093/jxb/err065 21414961 10.1093/jxb/err065

[71] Manos P, Steele K. Phylogenetic analyses of "higher" Hamamelididae based on plastid sequence data. Am J Bot. 1997; 84:1407–1407. 21708548

[72] Piredda R, Simeone MC, Attimonelli M, Bellarosa R, Schirone B. Prospects of barcoding the Italian wild dendroflora: oaks reveal severe limitations to tracking species identity. Mol Ecol Resour. 2011; 11:72–83. doi: 10.1111/j.1755-0998.2010.02900.x 21429102 10.1111/j.1755-0998.2010.02900.x

[73] Ueno S, Taguchi Y, Tsumura Y. Microsatellite markers derived from Quercus mongolica var. crispula (Fagaceae) inner bark expressed sequence tags. Genes Genet Sys. 2008; 83:179–187. 10.1266/ggs.83.179 18506101

[74] Steinkellner H, Lexer C, Turetschek E, Glössl J. Conservation of (GA)n microsatellite loci between Quercus species. Mol Ecol. 1997; 6:1189–1194. 10.1023/a:1005736722794 9154990

[75] Thompson JD, Gibson TJ, Plewniak F, Jeanmougin F, Higgins DG. The CLUSTAL_X windows interface: flexible strategies for multiple sequence alignment aided by quality analysis tools. Nucleic Acids Res. 1997; 25:4876–4882. 9396791 10.1093/nar/25.24.4876 PMC147148

[76] Hall TA. BioEdit: a user-friendly biological sequence alignment editor and analysis program for Windows 95/98/NT. Nucl acid S. 1999; 41:95–98.

[77] Rambaut A. Figtree 1.4.0. 2012. http://tree.bio.ed.ac.uk/software/figtree/

[78] Tamura K, Peterson D, Peterson N, Stecher G, Nei M, Kumar S. MEGA5: molecular evolutionary genetics analysis using maximum likelihood, evolutionary distance, and maximum parsimony methods. Mol Biol Evol. 2011; 28:2731–2739. doi: 10.1093/molbev/msr121 21546353 10.1093/molbev/msr121 PMC3203626

[79] http://www.ebi.ac.uk/Tools/msa/mafft/.

[80] Posada D, Crandall KA. Modeltest: testing the model of DNA substitution. Bioinformatics. 1998; 14:817–818. 9918953 10.1093/bioinformatics/14.9.817

[81] Posada D, Buckley TR. Model selection and model averaging in phylogenetics: advantages of Akaike information criterion and Bayesian approaches over likelihood ratio tests. Systematic biology. 2004; 53:793–808. doi: 10.1080/10635150490522304 15545256 10.1080/10635150490522304

[82] Ronquist F, Huelsenbeck JP. MrBayes 3: Bayesian phylogenetic inference under mixed models. Bioinformatics. 2003; 19:1572–1574. 12912839 10.1093/bioinformatics/btg180

[83] Yang Z, Wong WS, Nielsen R. Bayes empirical Bayes inference of amino acid sites under positive selection. Mol Biol Evol. 2005; 22: 1107–1118. doi: 10.1093/molbev/msi097 15689528 10.1093/molbev/msi097

[84] Nielsen R, Yang Z. Likelihood models for detecting positively selected amino acid sites and applications to the HIV-1 envelope gene. Genetics. 1998; 148:929–936. 9539414 10.1093/genetics/148.3.929 PMC1460041

[85] Yang Z, Nielsen R, Goldman N, Pedersen AMK. Codon-substitution models for heterogeneous selection pressure at amino acid sites. Genetics. 2000; 155:431–449. 10790415 10.1093/genetics/155.1.431 PMC1461088

[86] Fares MA, McNally D. CAPS: coevolution analysis using protein sequences. Bioinformatics. 2006; 22:2821–2822. doi: 10.1093/bioinformatics/btl493 17005535 10.1093/bioinformatics/btl493

[87] R Development Core Team (2008). R: A language and environment for statistical computing. R Foundation for Statistical Computing, Vienna, Austria ISBN 3-900051-07-0, URL http://www.R-project.org.

[88] Breiman L, Friedman JH, Olshen RA, Stone CJ. Classification and Regression Trees. Chapmann and Hall;1984.

[89] Manos PS, Doyle JJ, Nixon KC. Phylogeny, biogeography, and processes of molecular differentiation in *Quercus* subgenus *Quercus* (Fagaceae). Mol Phylogenet Evol. 1999; 12:333–349. doi: 10.1006/mpev.1999.0614 10413627 10.1006/mpev.1999.0614

[90] Yang Z. PAML 4: phylogenetic analysis by maximum likelihood. Mol Biol Evol. 2007; 24:1586–1591. doi: 10.1093/molbev/msm088 17483113 10.1093/molbev/msm088

[91] Jordan DB, Ogren WL. Species variation in the specificity of ribulose biphosphate carboxylase/oxygenase. Nature. 1981; 291:513–515.

[92] Ott CM, Smith BD, Portis AR, Spreitzer RJ. Activase Region on Chloroplast Ribulose-1,5-bisphosphate Carboxylase/Oxygenase nonconservative substitution in the large subunit alters species specificity of protein interaction. J Biol Chem. 2000; 275:26241–26244. doi: 10.1074/jbc.M004580200 10858441 10.1074/jbc.M004580200

[93] Portis AR Jr. Rubisco activase—Rubisco's catalytic chaperone. Photosynth Res. 2003; 75:11–27. doi: 10.1023/A:1022458108678 16245090 10.1023/A:1022458108678

[94] Du YC, Spreitzer RJ. Suppressor mutations in the chloroplast-encoded large subunit improve the thermal stability of wild-type ribulose-1, 5-bisphosphate carboxylase/oxygenase. J Biol Chem. 2000; 275:19844–19847. doi: 10.1074/jbc.M002321200 10779514 10.1074/jbc.M002321200

[95] Spreitzer RJ, Salvucci ME. Rubisco: structure, regulatory interactions, and possibilities for a better enzyme. Annu Rev Plant Biol. 2002; 53:449–475. doi: 10.1146/annurev.arplant.53.100301.135233 12221984 10.1146/annurev.arplant.53.100301.135233

[96] Knight S, Andersson I, Brändén CI. Crystallographic analysis of ribulose1,5-bisphosphate carboxylase from spinach at 2· 4 Å resolution: Subunit interactions and active site. J Mol Biol. 1990; 215:113–160. doi: 10.1016/S0022-2836(05)80100-7 2118958 10.1016/S0022-2836(05)80100-7

[97] Zhu XG, Jensen RG, Bohnert HJ, Wildner GF, Schlitter J. Dependence of catalysis and CO_2_/O_2_ specificity of Rubisco on the carboxy-terminus of the large subunit at different temperatures. Photosynth Res. 1998; 57:71–79.

[98] Niinemets Ü, Sack L. Structural determinants of leaf light-harvesting capacity and photosynthesis potentials In: Esser K, Lüttge UE, Beyschlag W, Murata J, editors. Progress in botany. Springer Verlag, Berlin; 2006 pp. 385–419.

[99] Kellogg E, Juliano N. The structure and function of Rubisco and their implications for systematic studies. Am J Bot. 1997; 84:413–413. 21708595

[100] Makowski M, Sobolewski E, Czaplewski C, Oldziej S, Liwo A, Scheraga HA. Simple physics-based analytical formulas for the potentials of mean force for the interaction of amino acid side chains in water. IV. Pairs of different hydrophobic side chains. J Phys Chem B. 2008; 112:11385–11395. doi: 10.1021/jp803896b 18700740 10.1021/jp803896b PMC2761002

