# Correction: Positively selected amino acid replacements within the RuBisCO enzyme of oak trees are associated with ecological adaptations

**DOI:** 10.1371/journal.pone.0188984

**Published:** 2017-12-11

**Authors:** Carmen Hermida-Carrera, Mario A. Fares, Ángel Fernández, Eustaquio Gil-Pelegrín, Maxim V. Kapralov, Arnau Mir, Arántzazu Molins, José Javier Peguero-Pina, Jairo Rocha, Domingo Sancho-Knapik, Jeroni Galmés

In the Funding section, the following information is missing: A. Mir and J. Rocha were partially funded by the Spanish research project DPI2015-67082-P (AEI/FEDER, UE).

The images for Figs 1 and 2 are incorrectly switched. The image that appears as Fig 1 should be Fig 2, and the image that appears as Fig 2 should be Fig 1. The figure captions appear in the correct order.

There are also errors in the figure captioned “*Quercus* large dataset Bayesian phylogram based on 158 *rbcL* sequences.” Please see the correct Figs 1 and 2 below.

**Fig 1:**
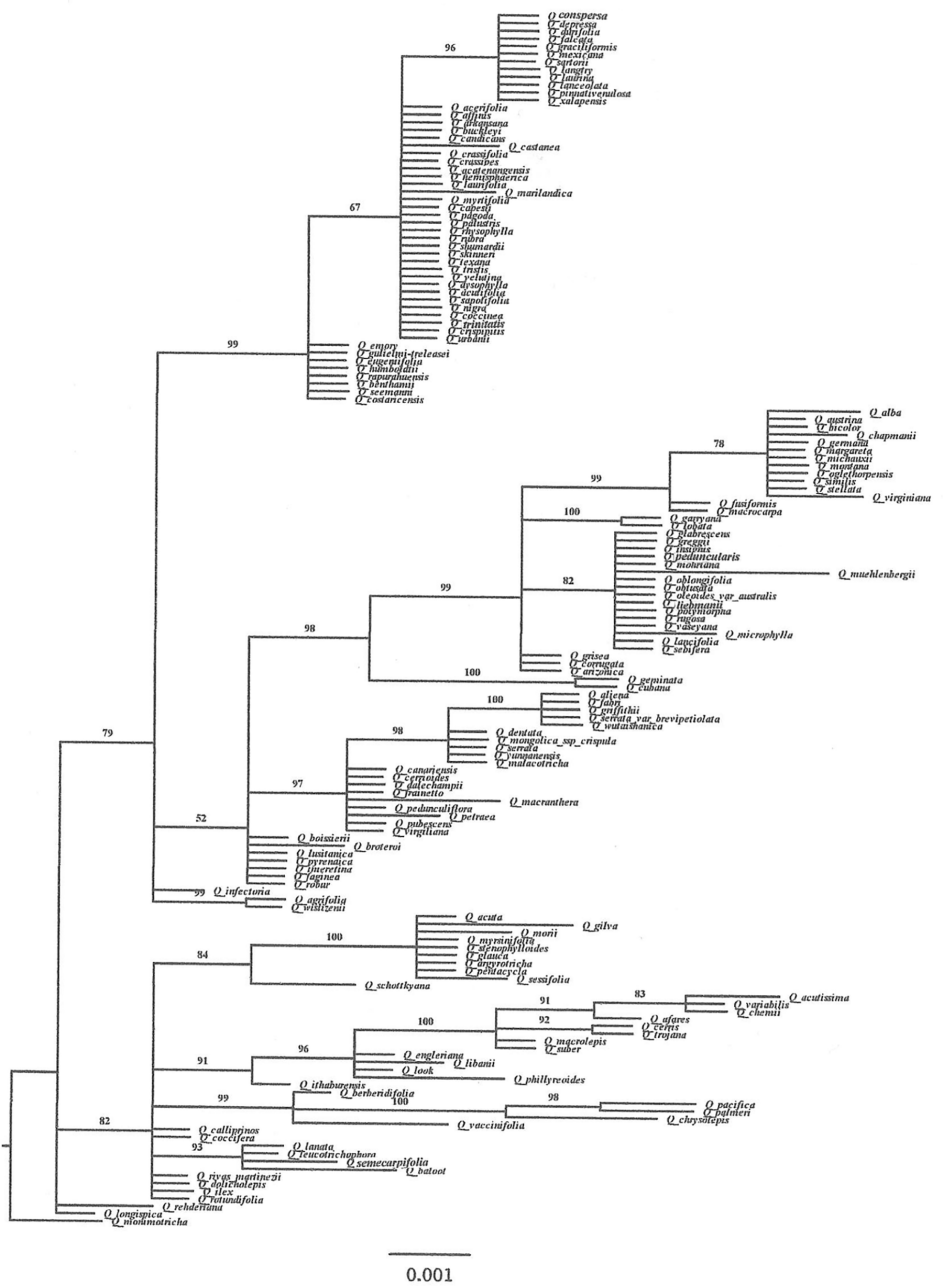
*Quercus* large dataset Bayesian phylogram based on 158 *rbcL* sequences. Numbers above branches correspond to Bayesian posterior probabilities. The figure was edited using FigTree Version 1.4.0 [77].

**Fig 2:**
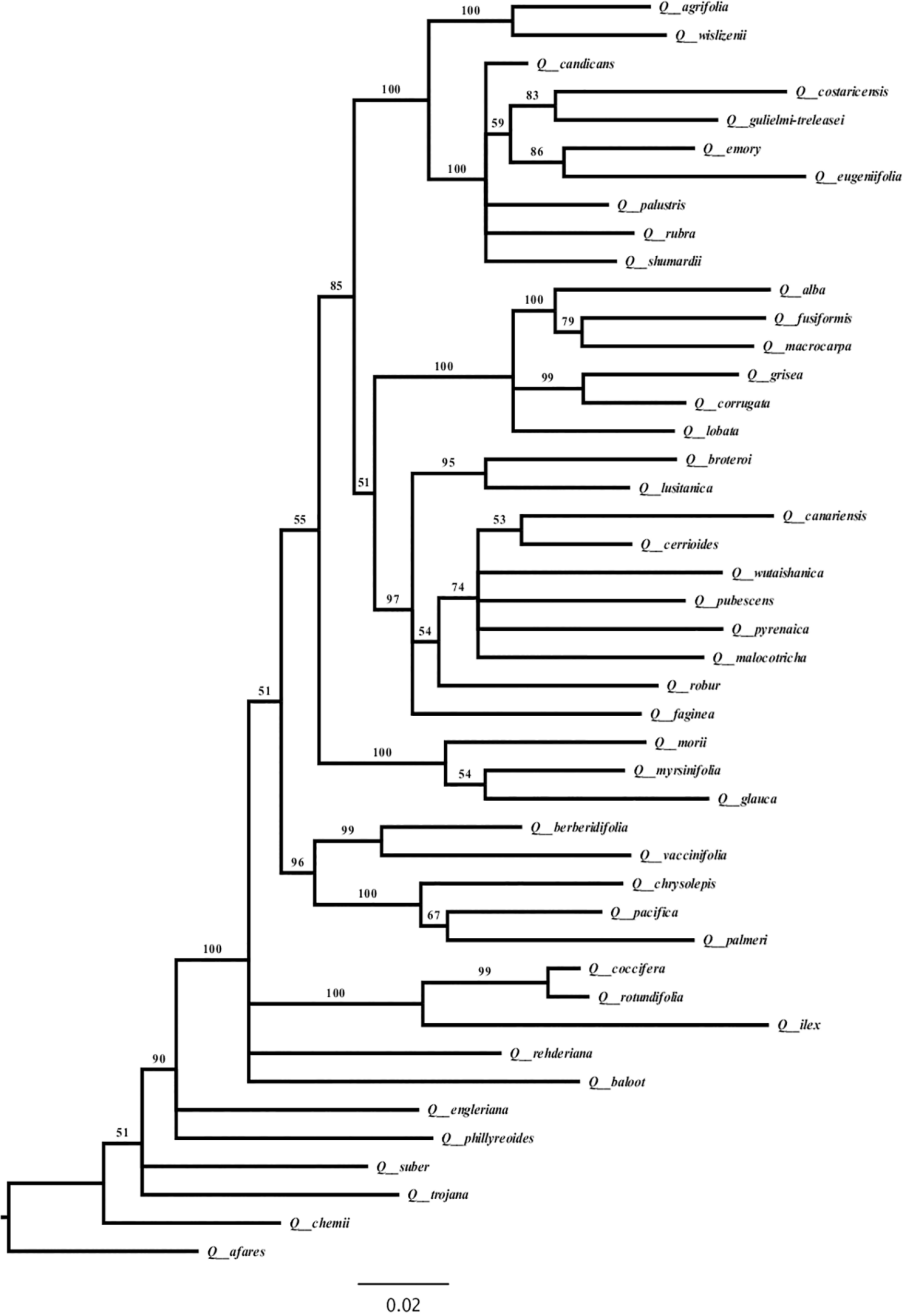
Quercus small dataset Bayesian phylogeny based on 45 sequences of rbcL, 43 matK and 42 microsatellites. Numbers above branches correspond to Bayesian posterior probabilities. The figure was edited using FigTree Version 1.4.0 [77].
