# Supplementary material for: Positively selected amino acid replacements within the RuBisCO enzyme of oak trees are associated with ecological adaptations
Source: PLoS One. 2017 Aug 31;12(8):e0183970. doi: 10.1371/journal.pone.0183970 (PMC5578625; doi:10.1371/journal.pone.0183970)
Supplement: S1 Table — The genus, subgenus and section are indicated along with information on the species distribution, climate, and leaf habit and density. Data on the geographic distribution and leaf habit were obtained from Govaerts et al. (1998) [64] and publicly available databases [65, 66, 67, 68]. The climate types were obtained by overlapping the species geographical distribution and the Köppen-Geiger world map of climate classification [69]. Fifteen different Köppen-Geiger types of climates were grouped into six: 1 = tropical, 2 = arid steppe, 3 = temperate with dry winter and hot or warm summer, 4 = temperate with dry summer and hot or warm summer, 5 = temperate or cold without dry season and hot or warm summer, 6 = cold with dry summer and hot or warm summer. The species leaf density was calculated from leaf thickness and leaf mass area (LMA) measurements. The three columns on the right correspond with [1] classification. (PDF) [file pone.0183970.s001.pdf]

S1 Table.

Family	Genus	Subgenus	Section	Species	Geographic distribution	Climate	Leaf habit	Leaf density (kg m^{-3})	Infrageneric Group	Genus	Family
Fagaceae	*Quercus*	*Quercus*	*Quercus*	*alba*	North America	5	Deciduous	1095.8	*Quercus*	*Quercus*	*Fagaceae*
				aliena	Asia	3	Deciduous	832.7			
				arizonica	North America	4	Evergreen	429.5			
				austrina	North America	5	Deciduous	466.2			
				berberidifolia	North America	4	Evergreen	822.3			
				bicolor	North America	5	Deciduous	1047.6			
				boissierii	Eurasia	4	Deciduous	955.8			
				broteroi	Eurasia	4	Deciduous	922.5			
				canariensis	Eurasia	4	Deciduous	1084.3			
				cerrioides	Eurasia	5	Deciduous	835.6			
				chapmanii	North America	5	Semi-evergreen	813.4			
				cubana	Central America	1	Evergreen	340.3			
				dalechampii	Eurasia	5	Deciduous	888.8			
				dentata	Asia	3	Deciduous	825.5			
				engleriana	Asia	3	Semi-evergreen	495.1			
				fabri	Asia	3	Deciduous	905.7			
				faginea	Eurasia	5	Deciduous	785.4			
				frainetto	Eurasia	5	Deciduous	1013.0			
				fusiformis	North America	2	Evergreen	631.0			
				garryana	North America	4	Deciduous	1063.5			
				geminata	North America	5	Semi-evergreen	755.3			
				germana	Eurasia	3	Semi-evergreen	927.3			
				glabrescens	Eurasia	3	Deciduous	501.9			

greggii	Eurasia	5	Evergreen	812.8
griffithii	Asia	3	Deciduous	1005.9
grisea	North America	4	Semi-evergreen	416.8
imeretina	Eurasia	5	Deciduous	472.4
infectoria	Eurasia	6	Deciduous	363.8
insignis	Central America	1	Deciduous	862.4
lanata	Asia	3	Evergreen	497.8
lancifolia	Central America	1	Deciduous	501.6
leucotrichophora	Asia	3	Evergreen	804.0
lobata	North America	4	Deciduous	1107.5
lusitanica	Eurasia	5	Deciduous	838.4
macranthera	Eurasia	6	Deciduous	841.7
macrocarpa	North America	5	Deciduous	938.7
malacotricha	Asia	3	Deciduous	973.6
margareta	North America	5	Deciduous	399.1
peduncularis	Central America	4	Evergreen	381.8
michauxii	North America	5	Deciduous	1099.8
microphylla	Central America	3	Deciduous	453.4
mohriana	North America	6	Evergreen	419.3
mongolica ssp. *crispula*	Asia	3	Deciduous	854.4
montana	North America	5	Deciduous	931.9
muehlenbergii	North America	5	Deciduous	850.2
oblongifolia	North America	4	Evergreen	372.0
obtusata	Central America	4	Evergreen	407.4
oglethorpensis	North America	5	Deciduous	1021.1
oleoides var. *australis*	Central America	1	Evergreen	570.2

	pacifica	North America	4	Evergreen	405.4	
	liebmanii	Central America	1	Semi-evergreen	446.1	
	pedunculiflora	Eurasia	5	Deciduous	799.3	
	petraea	Eurasia	5	Deciduous	798.2	
	corrugata	Central America	1	Deciduous	412.7	
	polymorpha	Central America	2	Deciduous	578.7	
	pubescens	Eurasia	5	Deciduous	906.0	
	pyrenaica	Eurasia	5	Deciduous	943.1	
	robur	Eurasia	5	Deciduous	903.6	
	rugosa	Central America	4	Semi-evergreen	733.3	
	sebifera	Central America	3	Evergreen	500.2	
	serrata	Asia	5	Deciduous	660.9	
	serrata var. *brevipetiolata*	Asia	5	Deciduous	977.7	
	similis	North America	5	Deciduous	977.1	
	stellata	North America	5	Deciduous	1013.9	
	vaseyana	North America	6	Semi-evergreen	895.9	
	virgiliana	North America	5	Semi-evergreen	461.7	
	virginiana	North America	5	Semi-evergreen	607.9	
	wutaishanica	Asia	3	Deciduous	490.9	
	yunnanensis	Asia	3	Deciduous	1189.3	
Lobatae	*conspersa*	Central America	3	Evergreen	430.0	*Lobatae*
	acerifolia	North America	5	Deciduous	567.0	
	acutifolia	Central America	4	Semi-evergreen	510.1	
	affinis	Central America	3	Evergreen	668.7	
	agrifolia	North America	4	Evergreen	953.3	
	arkansana	North America	5	Deciduous	1150.8	

benthamii	Central America	1	Evergreen	594.0
buckleyi	North America	5	Deciduous	1028.0
candicans	Central America	4	Evergreen	405.0
capesii	Central America	5	Deciduous	416.8
castanea	Central America	4	Evergreen	849.0
coccinea	North America	5	Deciduous	533.8
costaricensis	Central America	5	Evergreen	886.8
crassifolia	Central America	4	Semi-evergreen	448.3
crassipes	Central America	3	Evergreen	756.1
acatenangensis	Central America	1	Evergreen	489.2
crispipilis	Central America	3	Semi-evergreen	443.2
trinitatis	Central America	3	Evergreen	539
depressa	Central America	3	Evergreen	733.7
durifolia	Central America	4	Evergreen	927.5
dysophylla	Central America	3	Deciduous	255.8
emory	Central America	4	Evergreen	740.1
eugeniifolia	Central America	3	Deciduous	320.8
falcata	North America	5	Deciduous	946.7
graciliformis	Central America	2	Evergreen	392.2
gulielmi-treleasei	Central America	1	Evergreen	894.1
hemisphaerica	North America	5	Evergreen	845.4
humboldtii	Central America	1	Evergreen	503.8
lanceolata	Central America	4	Evergreen	521.6
langtry	Central America	2	Evergreen	311.4
laurifolia	North America	5	Deciduous	516.4
laurina	Central America	3	Evergreen	767.4

	marilandica	North America	5	Deciduous	967.2	
	mexicana	Central America	3	Evergreen	730.1	
	myrtifolia	North America	5	Evergreen	788.5	
	nigra	North America	5	Deciduous	933.1	
	pagoda	North America	5	Deciduous	1112.2	
	palustris	North America	5	Deciduous	927.1	
	pinnativenulosa	Central America	3	Evergreen	502.3	
	rapurahuensis	Central America	1	Evergreen	945.2	
	rhysophylla	Central America	2	Evergreen	908.5	
	rubra	North America	5	Deciduous	1077.4	
	sapotifolia	Central America	1	Evergreen	400.0	
	sartorii	Central America	3	Evergreen	469.8	
	seemanni	Central America	1	Deciduous	865.4	
	shumardii	North America	5	Deciduous	1062.5	
	skinneri	Central America	1	Evergreen	510.9	
	texana	North America	5	Deciduous	1077.5	
	tristis	Central America	1	Evergreen	972.2	
	urbanii	Central America	4	Deciduous	386.6	
	velutina	North America	5	Deciduous	922.1	
	wislizenii	North America	4	Evergreen	691.8	
	xalapensis	Central America	3	Evergreen	778.5	
Cerris	*acutissima*	Asia	3	Deciduous	927.6	*Cerris*
	afares	Eurasia	6	Deciduous	945.6	
	cerris	Eurasia	5	Deciduous	883.8	
	chemii	Asia	5	Deciduous	473.3	
	ithaburensis	Eurasia	4	Semi-evergreen	429.5	

		libanii	Eurasia	6	Deciduous	903.4	
		look	Eurasia	4	Semi-evergreen	583.5	
		macrolepis	Eurasia	4	Deciduous	901.6	
		suber	Eurasia	4	Evergreen	769.9	
		trojana	Eurasia	6	Semi-evergreen	966	
		variabilis	Asia	3	Deciduous	906.6	
		baloot	Asia	6	Evergreen	276.8	*Ilex*
		calliprinos	Eurasia	4	Evergreen	796.4	
		coccifera	Eurasia	4	Evergreen	708.5	
		dolicholepis	Asia	3	Evergreen	493.8	
		ilex	Eurasia	4	Evergreen	865.2	
		longispica	Asia	3	Evergreen	1068.7	
		monimotricha	Asia	3	Evergreen	423.7	
		phillyreoides	Asia	5	Evergreen	788.2	
		rehderiana	Asia	3	Evergreen	817.9	
		rivas-martinezii	Eurasia	4	Evergreen	476	
		rotundifolia	Eurasia	4	Evergreen	736.3	
		semecarpifolia	Asia	3	Evergreen	409.3	
	Protobalanus	*chrysolepis*	North America	4	Evergreen	711.7	*Protobalanus*
		palmeri	North America	4	Evergreen	506.4	
		vaccinifolia	North America	6	Evergreen	516.1	
Cyclobalanopsis		*acuta*	Asia	5	Evergreen	903.8	*Cyclobalanopsis*
		argyrotricha	Asia	5	Semi-evergreen	514.9	
		gilva	Asia	5	Evergreen	863.4	
		glauca	Asia	3	Evergreen	830.6	
		morii	Asia	3	Evergreen	503.2	

		myrsinifolia	Asia	5	Evergreen	1089.0		
		pentacycla	Asia	3	Evergreen	494.9		
		schottkyana	Asia	3	Evergreen	695.4		
		sessifolia	Asia	5	Evergreen	479.1		
		stenophylloides	Asia	3	Evergreen	476.9		
	Fagus	*engleriana*	Asia	3	Deciduous	669.0	*Fagus*	
		grandifolia	North America	5	Deciduous	544.8		
		japonica	Asia	5	Deciduous	778.8		
		lucida	Asia	5	Deciduous	609.7		
		sylvatica	Eurasia	5	Deciduous	704.8		
		crenata	Asia	5	Deciduous	624.1		
	Castanea	*mollissima*	Asia	5	Deciduous	711.2	*Castanea*	
		pumila	North America	5	Deciduous	468.7		
		sativa	Eurasia	5	Deciduous	700.7		
	Castanopsis	*carlesi*	Asia	5	Evergreen	583.9	*Castanopsis*	
	Lithocarpus	*densiflorus*	North America	4	Evergreen	746.2	*Notholithocarpus*	
		hancei	Asia	5	Evergreen	462.9	*Lithocarpus*	
Nothofagaceae	*Nothofagus*	*antarctica*	South America	5	Deciduous	767.0	*Nothofagus*	
		menciazii	New Zealand	5	Evergreen	198.6		*Nothofagaceae*
		moorei	Australia	5	Evergreen	671.2		
		procera	South America	5	Deciduous	649.9		
