# Supplementary material for: Positively selected amino acid replacements within the RuBisCO enzyme of oak trees are associated with ecological adaptations
Source: PLoS One. 2017 Aug 31;12(8):e0183970. doi: 10.1371/journal.pone.0183970 (PMC5578625; doi:10.1371/journal.pone.0183970)
Supplement: S2 Table — To simplify the analysis, 15 different Köppen-Geiger types of climates were grouped into six: 1) tropical (including climates Af, Am and Aw according to Köppen-Geiger classification); 2) arid steppe (Bsh, Bsk); 3) temperate with dry winter and hot or warm summer (Cwa, Cwb); 4) temperate with dry summer and hot or warm summer (Csa, Csb); 5) temperate or cold without dry season and hot or warm summer (Cfa, Cfb, Dfa, Dfb), and 6) cold with dry summer and hot or warm summer (Dsa, Dsb). (PDF) [file pone.0183970.s002.pdf]

S2 Table.

Köppen-Geiger classification		Climate groups	
Abreviation	Name		
Af	Tropical rainforest	1	Tropical
Am	Tropical monsoon		
Aw	Tropical savannah		
Bsh	Arid steppe hot	2	Arid steppe
Bsk	Arid steppe cold		
Cwa	Temperate dry winter hot summer	3	Temperate with dry winter and hot or warm summer
Cwb	Temperate dry winter warm summer		
Csa	Temperate dry summer hot summer	4	Temperate with dry summer and hot or warm summer
Csb	Temperate dry summer warm summer		
Cfa	Temperate without dry season hot summer	5	Temperate or cold without dry season and hot or warm summer
Cfb	Temperate without dry season warm summer		
Dfa	Cold without dry season hot summer		
Dfb	Cold without dry season warm summer		
Dsa	Cold dry summer hot summer	6	Cold with dry summer and hot or warm summer
Dsb	Cold dry summer warm summer		
