# Supplementary material for: Positively selected amino acid replacements within the RuBisCO enzyme of oak trees are associated with ecological adaptations
Source: PLoS One. 2017 Aug 31;12(8):e0183970. doi: 10.1371/journal.pone.0183970 (PMC5578625; doi:10.1371/journal.pone.0183970)
Supplement: S3 Table — (PDF) [file pone.0183970.s003.pdf]

S3 Table.

	Accession No.	
Species	*rbcL*	*matk*
Quercus alba	MF044819	MF044995
Quercus aliena	MF044820	
Quercus arizonica	MF044821	
Quercus austrina	MF044822	
Quercus berberidifolia	MF044823	MF044996
Quercus bicolor	MF044824	
Quercus boissierii	MF044825	
Quercus broteroi	MF044826	MF044997
Quercus canariensis	MF044827	MF044998
Quercus cerrioides	MF044828	MF045000
Quercus chapmanii	MF044829	
Quercus cubana	MF044830	
Quercus dalechampii	MF044831	
Quercus dentata	MF044832	
Quercus engleriana	MF044833	MF045005
Quercus fabri	MF044834	
Quercus faginea	MF044835	MF045006
Quercus frainetto	MF044836	
Quercus fusiformis	MF044837	MF045007
Quercus garryana	MF044838	
Quercus geminata	MF044839	
Quercus germana	MF044840	
Quercus glabrescens	MF044841	
Quercus greggii	MF044842	
Quercus griffithii	MF044843	
Quercus grisea	MF044844	MF045008
Quercus imeretina	MF044845	
Quercus infectoria	MF044846	
Quercus insignis	MF044847	
Quercus lanata	MF044848	
Quercus lancifolia	MF044849	
Quercus leucotrichophora	MF044850	
Quercus lobata	MF044851	MF045011
Quercus lusitanica	MF044852	MF045012
Quercus macranthera	MF044853	
Quercus macrocarpa	MF044854	MF045013
Quercus malacotricha	MF044855	MF045031
Quercus margareta	MF044856	
Quercus peduncularis	MF044857	
Quercus michauxii	MF044858	
Quercus microphylla	MF044859	
Quercus mohriana	MF044860	

Quercus mongolica ssp crispula	MF044861	
Quercus montana	MF044862	
Quercus muehlenbergii	MF044863	
Quercus oblongifolia	MF044864	
Quercus obtusata	MF044865	
Quercus oglethorpensis	MF044866	
Quercus oleoides var.australis	MF044867	
Quercus pacifica	MF044868	MF045017
Quercus liebmanii	MF044869	
Quercus pedunculiflora	MF044870	
Quercus petraea	MF044871	
Quercus corrugata	MF044872	MF045016
Quercus polymorpha	MF044873	
Quercus pubescens	MF044874	MF045029
Quercus pyrenaica	MF044875	MF045019
Quercus robur	MF044876	MF045021
Quercus rugosa	MF044877	
Quercus sebifera	MF044878	
Quercus serrata	MF044879	
Quercus serrata var.brevipetiolata	MF044880	
Quercus similis	MF044881	
Quercus stellata	MF044882	
Quercus vaseyana	MF044883	
Quercus virgiliana	MF044884	
Quercus virginiana	MF044885	
Quercus wutaishanica	MF044886	MF045010
Quercus yunnanensis	MF044887	
Quercus conspersa	MF044888	
Quercus acerifolia	MF044889	
Quercus acutifolia	MF044890	
Quercus affinis	MF044891	
Quercus agrifolia	MF044892	MF044994
Quercus arkansana	MF044893	
Quercus benthamii	MF044894	
Quercus buckleyi	MF044895	
Quercus candicans	MF044896	MF044999
Quercus capesii	MF044897	
Quercus castanea	MF044898	
Quercus coccinea	MF044899	
Quercus costaricensis	MF044900	MF045003
Quercus crassifolia	MF044901	
Quercus crassipes	MF044902	
Quercus acatenangensis	MF044903	
Quercus crispipilis	MF044904	
Quercus trinitatis	MF044905	
Quercus depressa	MF044906	

Quercus durifolia	MF044907	
Quercus dysophylla	MF044908	
Quercus emory	MF044909	MF045004
Quercus eugeniifolia	MF044910	MF045028
Quercus falcata	MF044911	
Quercus graciliformis	MF044912	
Quercus gulielmi-treleasei]	MF044913	MF045009
Quercus hemisphaerica	MF044914	
Quercus humboldtii	MF044915	
Quercus lanceolata	MF044916	
Quercus langtry	MF044917	
Quercus laurifolia	MF044918	
Quercus laurina	MF044919	
Quercus marilandica	MF044920	
Quercus mexicana	MF044921	
Quercus myrtifolia	MF044922	
Quercus nigra	MF044923	
Quercus pagoda	MF044924	
Quercus palustris	MF044925	MF045018
Quercus pinnativenulosa]	MF044926	
Quercus rapurahuensis]	MF044927	
Quercus rhysophylla	MF044928	
Quercus rubra	MF044929	MF045022
Quercus sapotifolia	MF044930	
Quercus sartorii	MF044931	
Quercus seemannii	MF044932	
Quercus shumardii	MF044933	MF045023
Quercus skinneri	MF044934	
Quercus texana	MF044935	
Quercus tristis	MF044936	
Quercus urbanii	MF044937	
Quercus velutina	MF044938	
Quercus wislizenii	MF044939	MF045026
Quercus xalapensis	MF044940	
Quercus acutissima	MF044941	
Quercus afares	MF044942	MF044993
Quercus cerris	MF044943	
Quercus chemii	MF044944	MF045030
Quercus ithaburensis	MF044945	
Quercus libanii	MF044946	
Quercus look	MF044947	
Quercus macrolepis	MF044948	
Quercus suber	MF044949	MF045024
Quercus trojana	MF044950	MF045025
Quercus variabilis	MF044951	
Quercus baloot	MF044952	MF045035

Quercus calliprinos	MF044953	
Quercus coccifera	MF044954	MF045002
Quercus dolicholepis	MF044955	
Quercus ilex	MF044956	MF045032
Quercus longispica	MF044957	
Quercus monimotricha	MF044958	
Quercus phillyreoides	MF044959	MF045034
Quercus rehderiana	MF044960	MF045020
Quercus rivas martinezii	MF044961	
Quercus rotundifolia	MF044962	MF045033
Quercus semecarpifolia	MF044963	
Quercus chrysolepis	MF044964	MF045001
Quercus palmeri	MF044965	
Quercus vaccinifolia	MF044966	
Quercus acuta	MF044967	
Quercus argyrotricha	MF044968	
Quercus gilva	MF044969	
Quercus glauca	MF044970	MF045027
Quercus morii	MF044971	MF045014
Quercus myrsinifolia	MF044972	MF045015
Quercus pentacycla	MF044973	
Quercus schottkyana	MF044974	
Quercus sessifolia	MF044975	
Quercus stenophylloides]	MF044976	
Fagus engleriana	MF044977	
Fagus grandifolia	MF044978	
Fagus japonica	MF044979	
Fagus lucida	MF044980	
Fagus sylvatica	MF044981	
Fagus crenata	MF044982	
Castanea mollissima	MF044983	
Castanea pumila	MF044984	
Castanea sativa	MF044985	
Castanopsis carlesi	MF044986	
Lithocarpus densiflorus	MF044987	
Lithocarpus hancei	MF044988	
Nothofagus antarctica	MF044989	
Nothofagus menciazii	MF044990	
Nothofagus moorei	MF044991	
Nothofagus procera	MF044992	
