# Supplementary material for: Positively selected amino acid replacements within the RuBisCO enzyme of oak trees are associated with ecological adaptations
Source: PLoS One. 2017 Aug 31;12(8):e0183970. doi: 10.1371/journal.pone.0183970 (PMC5578625; doi:10.1371/journal.pone.0183970)
Supplement: S4 Table — Variable sites identified when Quercus large dataset (158 species) were analyzed separately are marked in grey (9 variable sites and 21 haplotypes). Species marked with an asterisk were used to construct the Quercus small dataset (45 species) phylogeny based on rbcL, matK and SSRs. (PDF) [file pone.0183970.s004.pdf]

S4 Table.

Species	Haplotype	30	95	142	143	145	219	225	226	251	262	270	309	328	340	353	449	470	472	475
Q. conspersa, Q. acerifolia, Q. acutifolia, Q. affinis, Q. arkansana, Q. buckleyi, Q. candicans, Q. capesii, Q. castanea, Q. coccinea, Q. crassifolia, Q. crassipes, Q. acatenangensis, Q. crispipilis, Q. trinitatis, Q. depressa, Q. durifolia, Q. dysophylla, Q. falcata, Q. graciliformis, Q. hemisphaerica, Q. lanceolata, Q. langtry, Q. laurifolia, Q. laurina, Q. marilandica, Q. mexicana, Q. myrtifolia, Q. nigra, Q. pagoda, Q. palustris*, Q. pinnativenulosa, Q. rhysophylla, Q. rubra*, Q. sapotifolia, Q. sartorii,Q. shumardii*, Q. skinneri, Q. texana, Q. tristis, Q. urbanii, Q. velutina, Q. xalapensis*	1	Q	N	T	S	S	V	I	Y	I	V	L	I	A	E	Y	A	P	M	L
Q. acuta, Q. argyrotricha, Q. glauca, Q. macrolepis, Q. morii*, Q. myrsinifolia*, Q. pentacycla, Q. sessifolia, Q. stenophylloides, Q. suber*, C. mollissima*	2	Q	N	T	S	S	L	I	Y	I	A	L	I	A	E	Y	A	P	M	L
Q. acutissima, Q. afares, Q. chemii*, Q. variabilis*	3	Q	N	T	S	S	V	I	Y	I	A	L	I	A	E	Y	A	P	M	L
L. densiflorus, Q. agrifolia, Q. wislizenii**	4	Q	S	T	S	S	L	I	Y	I	V	L	I	S	E	Y	A	P	M	L
Q. alba, Q. austrina, Q. bicolor, Q. chapmanii, Q. germana, Q. margareta, Q. michauxii, Q. montana, Q. oglethorpensis, Q. similis, Q. stellate, Q. virginiana*	5	Q	S	T	S	S	L	I	F	I	V	L	M	A	E	Y	A	P	M	L
Q. aliena, Q. fabri, Q. griffithii, Q. serrata var. *brevipetiolata, Q. wutaishanica**	6	Q	S	T	S	S	L	I	Y	M	V	L	I	A	E	Y	A	P	M	L
Q. baloot, Q. berberidifolia*, Q. calliprinos, Q. chrysolepis*, Q. coccifera*, Q. dolicholepsis, Q. engleriana*, Q. ilex*, Q. ithaburensis, Q. lanata, Q. leucotrichophora, Q. look, Q. pacifica*, Q. palmeri*, Q. rivas martinezii, Q. rotundifolia*, Q. schottkyana, Q. semecarpifolia*	7	Q	N	T	S	S	L	I	Y	I	A	L	I	S	E	Y	A	P	M	L
Q. boissierii, Q. broteroi, Q. faginea*, Q. imeretina, Q. lusitanica*, Q. pyrenaica*, Q. robur*, C. sativa*	8	Q	N	T	S	S	L	I	Y	I	V	L	I	A	E	Y	A	P	M	L

Q. canariensis , Q. cerrioides*, Q. dalechampii, Q. dentata, Q. frainetto. Q. malocotricha*, Q. mongolica ssp crispula, Q. pedunculiflora, Q. petraea, Q. pubescens*, Q. serrata, Q. virgiliana, Q. yunnanensis*	9	Q	S	T	S	S	L	I	Y	I	V	L	I	A	E	Y	A	P	M	L
*Q. cerris, Q. trojana**	10	Q	S	T	S	S	L	I	Y	I	A	L	I	A	E	Y	A	P	M	L
Q. benthamii, Q. costaricensis, Q. emory*, Q. eugeniifolia*, Q. gulielmi-treleasei*,Q. humboldtii, Q. rapurahuensis, Q. seemannii*	11	Q	N	T	S	S	V	I	Y	I	V	L	I	S	E	Y	A	P	M	L
Q. glabrescens , Q. greggii, Q. insignis, Q. lancifolia, Q. peduncularis, Q. microphylla, Q. mohriana, Q. oblongifolia, Q. obtusata, Q. oleoides var australis, Q. liebmanii, Q. polymorpha, Q. rugosa, Q. sebifera, Q. vaseyana	12	Q	N	T	S	S	V	I	F	I	V	L	I	A	E	Y	A	P	M	L
Q. fusiformis, Q. macrocarpa**	13	Q	N	T	S	S	L	I	F	I	V	L	M	A	E	Y	A	P	M	L
Q. arizonica, Q. garryana, Q. grisea, Q. lobata*, Q. corrugata**	14	Q	N	T	S	S	L	I	F	I	V	L	I	A	E	Y	A	P	M	L
Q. cubana, Q.geminata	15	Q	N	T	S	S	L	I	Y	I	V	L	M	A	E	Y	A	P	M	L
Q. gilva	16	Q	N	T	S	S	L	I	Y	I	A	L	I	A	E	Y	A	P	T	V
*Q. infectoria, Q. longispica, Q. monimotrica, Q. rehderiana**	17	Q	N	T	S	S	L	I	Y	I	V	L	I	S	E	Y	A	P	M	L
Q. macranthera	18	Q	S	T	S	S	L	I	Y	I	V	L	I	A	E	Y	A	P	T	V
*C. pumila ,Q. libanii, Q. vaccinifolia**	19	Q	S	T	S	S	L	I	Y	I	A	L	I	S	E	Y	A	P	M	L
*Q. phillyreoides**	20	Q	N	T	S	S	I	I	Y	I	V	L	I	S	E	Y	A	P	M	L
Q. muehlenbergii	21	Q	N	T	S	S	I	I	F	M	V	L	I	A	E	Y	A	P	M	L
F. crenata, F. engleriana, F. japonica, F. lucida, F. grandifolia	23	E	S	T	S	A	L	L	Y	I	V	L	I	S	E	Y	S	P	M	L
F. sylvatica	24	E	S	T	S	A	L	I	Y	I	V	L	I	S	E	Y	S	P	M	L
C. carlesi	25	Q	S	T	S	S	V	I	Y	I	V	L	I	A	E	Y	A	P	M	L
L. hancei	26	Q	N	P	A	A	V	I	Y	I	A	L	M	A	E	Y	A	P	M	L
N. antarctica	27	E	T	P	A	V	L	I	Y	I	V	I	I	A	D	F	S	E	M	L
N. menciazii	28	E	N	P	A	V	L	L	Y	I	V	I	M	A	D	F	S	E	M	L
N. moorei	29	E	N	P	A	V	L	I	Y	I	V	I	M	A	D	F	S	E	M	L

N. procera	30	E	T	P	A	A	L	I	Y	I	V	I	I	A	D	F	S	E	M	L
