# Supplementary material for: Positively selected amino acid replacements within the RuBisCO enzyme of oak trees are associated with ecological adaptations
Source: PLoS One. 2017 Aug 31;12(8):e0183970. doi: 10.1371/journal.pone.0183970 (PMC5578625; doi:10.1371/journal.pone.0183970)
Supplement: S5 Table — Significant correlation coefficients are shown (p < 0.001). Mean D1 and Mean D2 values correspond to the mean distance calculated for each coevolving position based on BLOSUM as calculated in the method of Fares and Travers (2006) [1]. The bootstraps are based on 100 resampling, the confidence level associated with the pair that coevolves (values greater than 75% are significant after a non-parametric resampling over the tree). Atomic distances were calculated from 3D crystal structure wherever available by measuring the average Euclidean distance between atoms of two amino acids (Å). Atomic distances are not used here as evidence of coevolution but rather as additional supporting information in the identification of functional and structural coevolution. A test for variability in hydrophobicity and molecular weight has been also conducted giving as a result the pair 30–340, 142–470 and 270–309. n.a. not available. (PDF) [file pone.0183970.s005.pdf]

S5 Table.

Coevolving pairs		Correlation	Mean D1	Mean D2	Bootstrap value	Pairwise atomic distance	Property maintained
30	145	0.5	0.2	0.5	1.0	21.5	n.a.
30	225	0.5	0.2	0.2	1.0	55.0	n.a.
30	270	0.7	0.2	0.1	1.0	34.6	n.a.
30	340	0.7	0.2	0.2	1.0	30.4	Molecular Weight
30	353	0.7	0.2	0.1	1.0	20.4	n.a.
30	449	1.0	0.2	0.1	1.0	60.2	n.a.
30	470	0.7	0.2	0.8	1.0	40.9	n.a.
95	309	0.3	1.7	0.5	0.9	20.1	n.a.
142	145	0.4	0.7	0.5	1.0	5.0	n.a.
142	270	0.2	0.7	0.1	1.0	24.4	n.a.
142	309	0.2	0.7	0.5	1.0	26.0	n.a.
142	340	0.2	0.7	0.2	1.0	26.0	n.a.
142	353	0.1	0.7	0.1	1.0	11.5	n.a.
142	470	0.2	0.7	0.8	1.0	38.1	Molecular Weight
143	145	0.3	0.1	0.5	0.9	5.6	n.a.
145	270	0.7	0.5	0.1	0.9	20.1	n.a.
145	340	0.7	0.5	0.2	0.9	25.5	n.a.
145	353	0.7	0.5	0.1	0.9	13.5	n.a.
145	470	0.7	0.5	0.8	0.9	36.3	n.a.
270	309	0.1	0.1	0.5	1.0	10.9	Hidrophobicity and Molecular Weight
270	340	1.0	0.1	0.2	1.0	24.2	n.a.
270	353	1.0	0.1	0.1	1.0	26.8	n.a.
270	470	1.0	0.1	0.8	1.0	25.4	n.a.

309	340	0.2	0.5	0.2	1.0	18.9	n.a.
309	470	0.1	0.5	0.8	1.0	21.0	n.a.
340	353	1.0	0.2	0.1	1.0	17.7	n.a.
340	470	1.0	0.2	0.8	1.0	15.8	n.a.
353	470	1.0	0.1	0.8	1.0	32.5	n.a.
472	475	1.0	0.3	0.1	1.0	5.2	n.a.
